# Nanomaterials Enhanced Sonodynamic Therapy for Multiple Tumor Treatment

**DOI:** 10.1007/s40820-025-01666-8

**Published:** 2025-02-24

**Authors:** Mengyao Yang, Xin Wang, Mengke Peng, Fei Wang, Senlin Hou, Ruirui Xing, Aibing Chen

**Affiliations:** 1 College of Chemical and Pharmaceutical Engineering, Hebei University of Science and Technology, Shijiazhuang, 050018 People’s Republic of China; 2 State Key Laboratory of Biopharmaceutical Preparation and Delivery, Institute of Process Engineering, Chinese Academy of Sciences, Beijing, 100190 People’s Republic of China; 3 The Second Hospital of Hebei Medical University, Shijiazhuang, 050000 People’s Republic of China

**Keywords:** Sonodynamic therapy, Nanosonosensitizers, Tumor accumulation, Surmounting the hypoxia, Orthotopic tumor

## Abstract

Sonodynamic therapy (SDT) as an emerging modality for malignant tumors mainly involves in sonosensitizers and low-intensity ultrasound (US), which can safely penetrate the tissue without significant attenuation. SDT not only has the advantages including high precision, non-invasiveness, and minimal side effects, but also overcomes the limitation of low penetration of light to deep tumors. The cytotoxic reactive oxygen species can be produced by the utilization of sonosensitizers combined with US and kill tumor cells. However, the underlying mechanism of SDT has not been elucidated, and its unsatisfactory efficiency retards its further clinical application. Herein, we shed light on the main mechanisms of SDT and the types of sonosensitizers, including organic sonosensitizers and inorganic sonosensitizers. Due to the development of nanotechnology, many novel nanoplatforms are utilized in this arisen field to solve the barriers of sonosensitizers and enable continuous innovation. This review also highlights the potential advantages of nanosonosensitizers and focus on the enhanced efficiency of SDT based on nanosonosensitizers with monotherapy or synergistic therapy for deep tumors that are difficult to reach by traditional treatment, especially orthotopic cancers.

## Highlights

- The main mechanisms and clinical potential of sonodynamic therapy are emphasized.
- The nanomaterials provide new prospects and development directions for enhancing the treatment of cancer.
- Recent developments of sonodynamic therapy for deep tumors that are difficult to reach by traditional treatment, especially orthotopic cancers.

## Graphical abstract

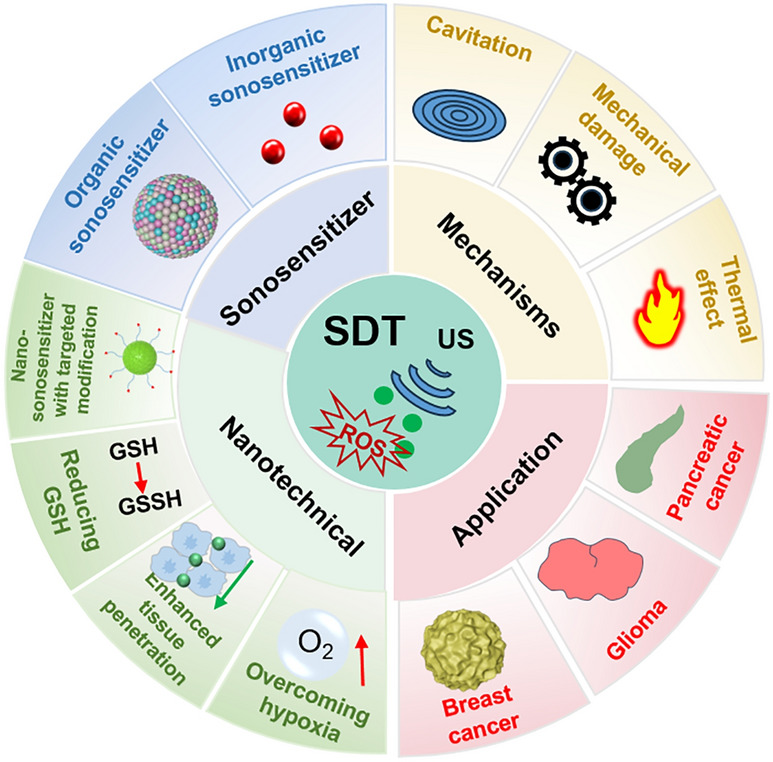

## Introduction

Nowadays, cancer has become one of the main causes of human death, and the prevalence rate is still on the rise every year [1–4]. Although the existing three major treatments, including surgery, chemotherapy, and radiotherapy, have achieved certain results in the treatment of cancer, serious adverse effects on normal tissues are inevitable for chemotherapy and radiotherapy as well as traumatic for surgery [5, 6]. Thus, it is urgent to develop an effective and precise non-invasive treatment. With the development of science and technology, phototherapy, including photodynamic therapy (PDT) and photothermal therapy (PTT), has developed a lot [7–9]. During PDT and PTT, the core component photosensitizer is excited under light irradiation with a suited wavelength and produces reactive oxygen species (ROS) and heat to kill tumor cells, respectively [10–14]. Phototherapy has a good application prospect owing to the merits of spatiotemporal selectivity, non-invasiveness, non-drug resistance, and less side effects [15–20]. However, because of the poor penetration depth of light, phototherapy is limited to the cure of lesion on the skin or surface [21–23].

In contrast, ultrasound (US) as one kind of mechanical waves has stronger tissue penetration, which is widely applied in the diagnosis of abdominal diseases [24, 25]. In recent years, it has made great progress in the fields of cardiac US, obstetrics and gynecology US, and endovascular US, so it has shown extensive application potential in the detection and treatment of deep-level tumors [26, 27]. In 1989, Umemura et.al found that the combination of US irradiation and hematoporphyrin showed effective antitumor effects in vivo and in vitro, which was attributed to the hematoporphyrin and was activated by US cavitation and generated the cytotoxic ROS [28]. By 1992, Umemura et al. proposed that this treatment was called “sonodynamic therapy” [29, 30]. As an emerging type of non-invasive modality, sonodynamic therapy (SDT) is developed based on PDT, mainly involving low-intensity US and sonosensitizers and having similar principles and function [31, 32]. Compared with other non-invasive treatment such as PDT and PTT, US possesses low tissue attenuation and deep penetration, allowing it to reach tissues up to more than 10 cm in depth [33]. This is significantly greater than the ≈1 cm penetration depth of near-infrared (NIR) light within tissues [34]. Consequently, SDT can address the limitation of traditional phototherapy in treating deep tumors. Furthermore, US is clinically recognized as a safe and effective imaging modality [35]. Hence, SDT holds great promise for clinical applications.

Although the therapeutic advantages of SDT have been confirmed in preclinical studies of diversified cancer cell lines, its further clinical application has been hindered since its mechanism has not been clarified [30]. For the sake of improving the anti-tumor therapeutic effect of SDT in vivo and promoting clinical transformation, it is necessary to have a deeper understanding of its basic mechanism and design a suitable sonosensitizer delivery system to make better use of the specific mechanism of SDT. The selection of sonosensitizers also performs a crucial role in the efficacy of SDT. Due to the shortcomings of traditional organic sonosensitizers such as low water solubility, poor tumor specificity, and early clearance, their retention in the tumor is limited, which affects the therapeutic effect [36–38]. Thanks to the rapid development of nanotechnology, various nanomaterials have been prepared into sonosensitizers or nanocarriers of sonosensitizers, greatly enhancing the accumulation of sonosensitizers. Owing to the rapid growth and abnormal metabolism of tumor cells, the tumor microenvironment (TME) is characterized by acidity, hypoxia, and elevated glutathione (GSH) [39, 40]. In addition, due to the limitations of a single treatment modality, SDT is usually synergistic with chemodynamic therapy (CDT), PTT, and immunotherapy to enhance the therapeutic effect [41–44].

**Scheme 1:**
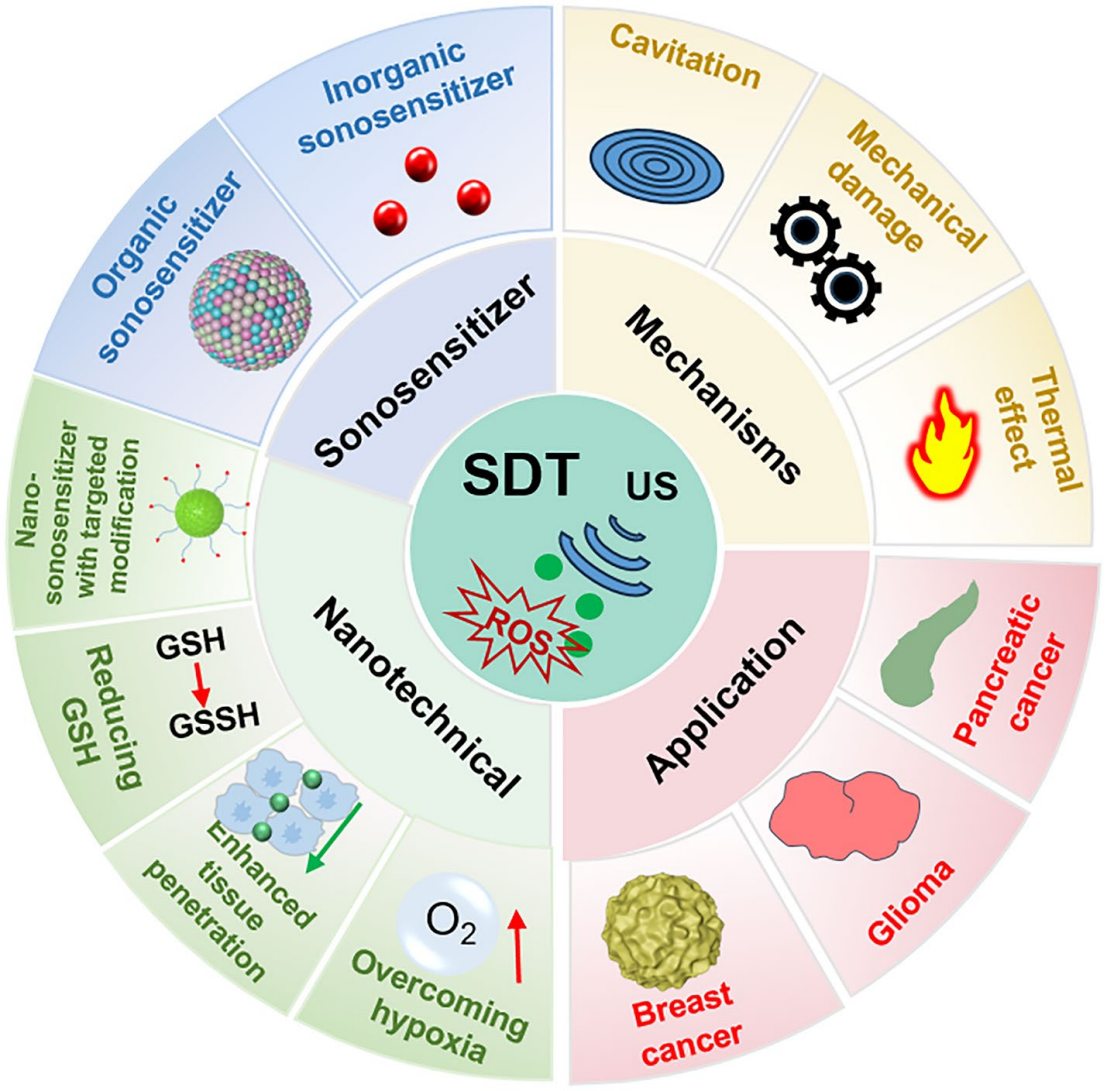
Overview of the mechanism, sonosensitizer, nanotechnical enhancement, and application of SDT

Therefore, in this review, we elucidate the mechanisms underlying SDT and conduct a comprehensive review of the latest advances in nanomaterials enhanced SDT, especially for orthotopic tumors, aiming to propel further progress in this field. Specifically, the main mechanism of SDT is briefly reviewed. Then, the reasonable design and manufacture of multi-functional sonosensitizers, including organic and inorganic sonosensitizers, are also introduced. The therapeutic efficacy of SDT is usually impeded by the constraints posed by the inherent structure of sonosensitizers and the tumor microenvironment, whereas advancements in nanotechnology provide a prospect to this challenge. This paper focuses on the effects of nanomaterials on enhancing SDT based on distinct functions, including increasing the accumulation of sonosensitizer at the lesion site, improving safety with image-guided precision therapy, reducing antioxidants (GSH) in the TME, and surmounting the hypoxic condition of the TME. In addition, the combination of SDT with other therapeutic modalities, such as CDT and immunotherapy, is also discussed, in particular for the treatment of orthotopic tumors (Scheme 1). As far as we know, this is the first to summarize the application of SDT in different types of orthotopic tumors. Finally, the prospects and challenges of this rapidly developing field will be addressed in the future.

## Mechanisms of Sonodynamic Therapy

**Fig. 1:**
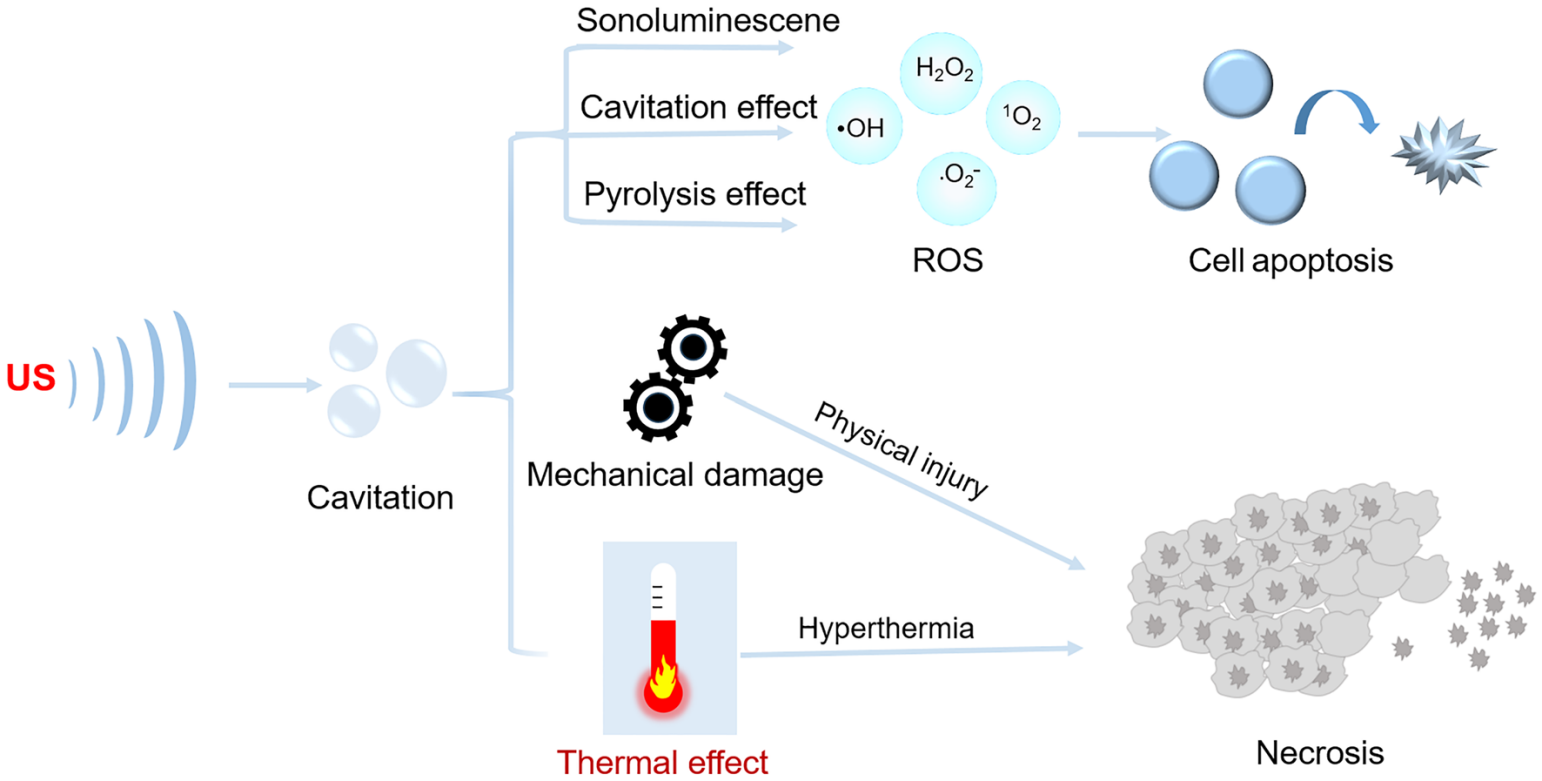
Main proposed mechanisms of SDT

In the realm of deep tumor therapy, SDT surpasses the penetration depth limitations faced by PDT. The high penetrating capability of low-intensity US allows it to penetrate deep tissues and activate the sonosensitizer at the target site, generating a substantial amount of ROS. This process exerts toxic effects on tumor cells while minimizing damage to adjacent normal tissues. When ROS scavenger was added to the experiment, the death rate of the cells decreased dramatically, which proved that ROS was the main cause of the death of the target cells [45]. However, the mechanism of ROS generation in SDT has not been exactly explained. At present, it is believed that the ROS generation in SDT can be generated mainly by cavitation effects, sonoluminescence, and pyrolysis under ultrasonic action [46]. Besides, mechanical damage and thermal effects caused by US can also cause damage to tumor cells (Fig. 1) [37, 47].

The cavitation effect is a unique phenomenon induced by the interaction between US and the water environment [42, 48]. Under the action of ultrasonic wave, the cavitation bubbles formed can oscillate, expand, and burst. Usually, under the action of low-intensity US, the liquid produces tiny bubbles that can contract and expand periodically, which is called stable cavitation. When the ultrasonic intensity is large enough, the dynamic change process of the bubble will be accelerated, forming inertial cavitation, where the bubble grows rapidly, expands, and then suddenly bursts. The shear force and shock wave generated by the above phenomena can cause mechanical damage to tumor cells. Another mechanical damage is caused by sonoporation [49], in which cavitation-induced shear forces and microflows can create temporary pores in the cell membrane. In addition, cavitation can produce reactive hydroxyl radicals and hydrogen atoms in the shear bubble. These free radicals and hydrogen atoms can produce new free radicals due to hydrodynamic reaction and stress with volatile molecules and enhance the production of hydroxyl radicals.

In addition to the cavitation effects, the sonoluminescence of US on sonosensitizers to produce cytotoxic ROS is considered to be another important mechanism to achieve SDT [50]. He et al. successfully detected sonoluminescence in vivo at low-frequency US (40 kHz) and low US pressure (0.2 MPa), proving the role of sonoluminescence in sonodynamic processes [51]. As one of the most common mechanisms for ROS production in SDT, sonoluminescence refers to the phenomenon of a particularly brief burst of light caused by the energy released by the fast burst of bubbles during cavitation [52]. Sonoluminescence can cause sonosensitizers to undergo photochemical reactions like photosensitizers and produce a large number of ROS, which further reveals that most photosensitizers exhibit the potential as sonosensitizers [53].

Another mechanism by which SDT induces ROS generation is called "pyrolysis" [54]. In the process of inertial cavitation, the energy released by the burst of the bubble can form a microenvironment of high temperature and pressure. Under high pressure and temperature, the sonosensitizer is thermally dissociated and decomposed into free radicals, which could further react with endogenous substances to form other ROS.

In general, US is utilized as the excitation source to sonosensitizers, producing ROS through different pathways, including cavitation, sonoluminescence, and pyrolysis, and eventually leads to apoptosis. In the meantime, mechanical action and thermal damage of SDT could also lead to cell necrosis [53, 55]. Due to the special cavitation effect of US and the resulting biological effect, the anti-tumor mechanism of SDT is more complex than PDT.

## Sonosensitizers

The sonochemical process with the participation of sonosensitizers plays an important role in the SDT process. Therefore, much effort has been made for SDT technology to develop new sonosensitizers with high sonosensitive activity. At present, the widely reported sonosensitizers could be divided into organic sonosensitizers and inorganic sonosensitizers [56, 57]. Similar to photosensitizers, organic sonosensitizers are mainly porphyrins, phthalocyanines, and their derivatives, such as protoporphyrin IX (PPIX), chlorin e6 (Ce6), and so on. In addition, rose bengal (RB), quinolones, natural products (like curcumin), some chemotherapy drugs (like doxorubicin), indocyanine green (ICG), and others have also been found to have sonosensitive effects [58–61], while inorganic sonosensitizers mainly refer to metal oxides (such as Ag_2_O, ZnO, TiO_2_, etc.) and piezoelectric materials (black phosphorus, barium titanate) [62–65].

### Organic Sonosensitizers

Porphyrin-based organic sonosensitizers were pioneers in the field of SDT. Notably, 5-aminolevulinic acid (5-ALA) has gained approval from Food and Drug Administration for glioma fluorescence-guided surgery. 5-ALA is an endogenous biochemical substance, after a series of enzymatic actions to produce a strong photosensitive PPIX, which generates ROS under light or US irradiation and triggers cell death. Beyond its promising clinical applications as a photosensitizer in PDT, 5-ALA is currently being studied clinically as sonosensitizers for SDT [66].

Organic sonosensitizers can be designed and synthesized according to specific needs owing to its strong structural adjustability [67]. Due to the advantages of good photostability and low biological toxicity, boron-dipyridine (BODIPY) dye is widely used in cancer imaging and treatment [68, 69]. However, there are few reports based on the employment of BODIPY as a sonosensitizer. Gao and coworkers synthesized four kinds of BODIPY dyes (BDP1-BDP4) with diverse structures according to the design principles of photosensitizer to study their potential application for SDT [70]. The authors compared the sonosensitivity of BDP1-BDP4. Among them, BDP4 has the best sonosensitive activity. In order to evaluate the sonosensitivity of BDP4, the authors studied the activity of BDP4 under different US conditions. BDP4 has higher sonosensitivity at a low frequency of 1 MHz, since the higher frequency produces smaller cavitation bubbles, and the cavitation nucleus does not have enough time to grow and induce cavitation effects timely. The sonosensitivity of BDP4 is also closely related to the intermittent transmission process of US. In light of their ideal electronic properties, boron-containing materials have been widely used in bioimaging and biomedicine. Chen et al. synthesized efficient organic sonosensitizers (BAnTh and BTeTh) based on a new strategy via doping triarylboron into acenethiophene scaffolds [71]. The connection of boron atoms to the linear acrylic system results in redshift absorption and emission compared to the precursor because of the lower lowest unoccupied molecular orbital (LUMO) energy and narrower band gap. Water-dispersed nanoparticles (BAnTh-NPs and BTeTh-NPs) were obtained by encapsulating BAnTh and BTeTh with the polymer DSPE-mPEG2000 and showed effective ·OH formation under ultrasonic irradiation. Photooxidation and theoretical studies show that the introduction of boron can significantly increase the organic carbon constant, which is conducive to efficient intersystem crossing. In addition, in vitro experiments have shown that SDT has high efficiency and low cytotoxicity and phototoxicity, which could avoid potential skin phototoxicity problems during cancer treatment. The in vivo treatment results of BTeTh-NPs showed good biocompatibility and significant tumor inhibition. More importantly, ROS generation studies have shown that, unlike traditional organic sonosensitivities, boron-doped acenaphthene overcomes the limitation of skin photosensitivity, providing hope for its further clinical conversion.

**Fig. 2:**
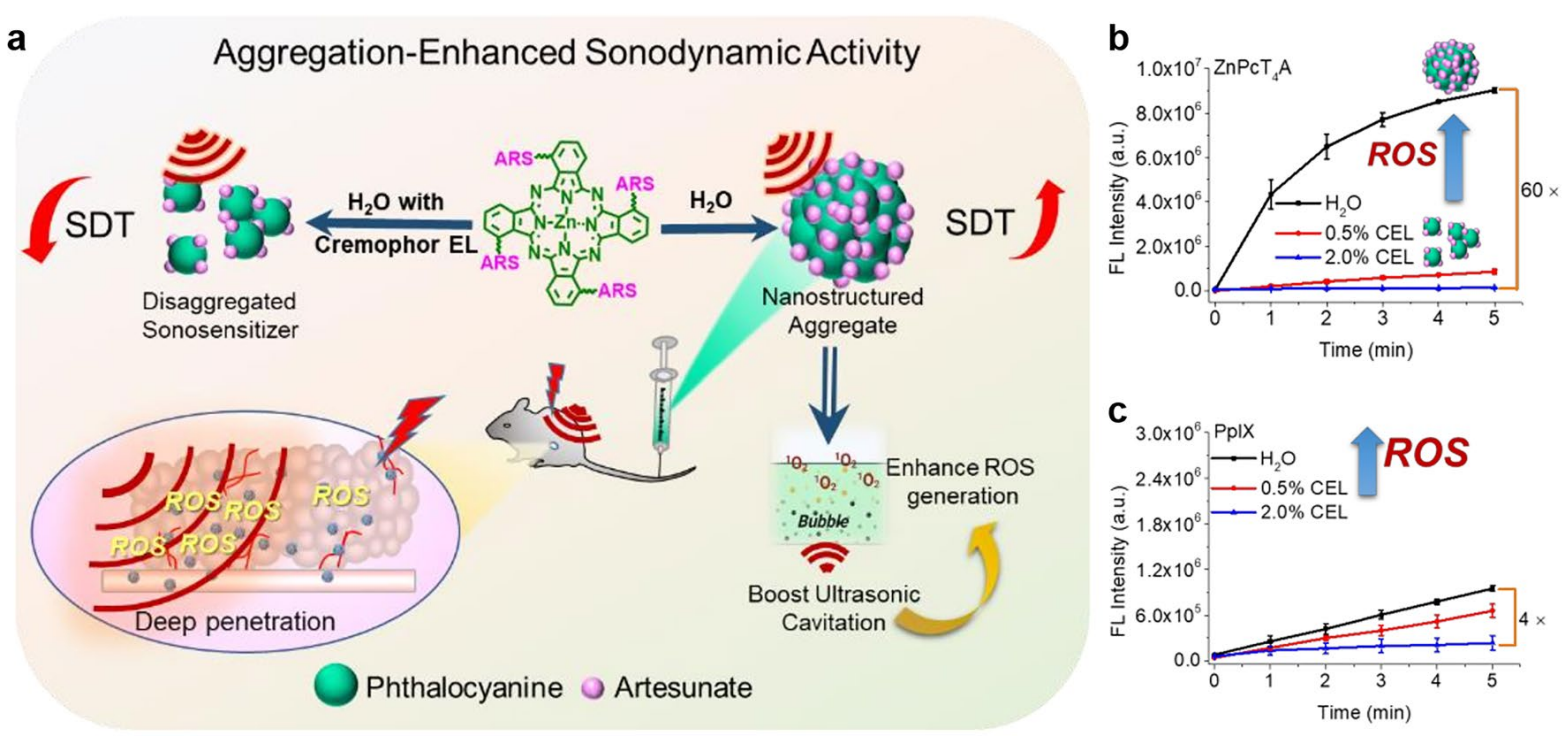
Phthalocyanine–artesunate couplets for SDT. **a** Conjugation of phthalocyanine with artesunate, exhibiting enhanced sonodynamic activity through aggregation. **b** and **c** ROS generation using 2’,7’-dichlorofluorescin diacetate (DCFHDA) as the fluorescence probe. Reproduced with permission [72]. Copyright 2021, John Wiley and Sons

However, due to problems caused by the structure of organic photosensitizer itself, such as the hydrophobicity, the direct application of simple organic sonosensitizer molecules often leads to poor therapeutic effect. The group of Huang designed three phthalocyanine–artesunate couplets (ZnPcT_1_A, ZnPcT_2_A, and ZnPcT_4_A) by coupling mono, di, and tetra-triethylene glycol-modified zinc (II) phthalocyanine and artesunate unit(s), separately (Fig. 2a) [72]. The sonodynamic ROS generation of these conjugates in the aggregate form was observably higher than that in the non-aggregate form. Among them, ZnPcT_4_A showed significant performance, and its ROS production in water (aggregated into nanostructure) was about 60 times higher than that in water with 2% Cremophor EL added (nanostructure disaggregated) (Fig. 2b). The aggregation ability of the sonosensitizer molecules and the particle size of the aggregates could affect the enhanced sonodynamic activity. What's more, this phenomenon can also be observed on other sonosensitizers, like ZnPcT_4_, Ce6, and PpIX (Fig. 2c). Thus, they propose that the aggregation-enhanced sonodynamic activity effect will be a common phenomenon in most organic sonosensitizers. In addition, both in vitro and in vivo studies have shown that ZnPcT_4_A aggregates have high biosafety and efficient SDT anti-cancer efficacy with tumor growth inhibition rate of up to 98%. The discovered aggregation-enhanced sonodynamic activity in this research fundamentally alters the conventional approach to avoiding aggregation in sonosensitizer design, thereby pioneering a fresh avenue for the advancement of highly efficient organic sonosensitizers.

### Inorganic Sonosensitizers

Although organic sonosensitizers have been proven to be effective in SDT, they have some shortcomings such as low bioavailability, poor stability, complex synthesis, and poor water solubility, which significantly reduce the ROS produced by US-activated sonosensitizers and restrain the treatment efficiency. In addition, most organic sonosensitizers originate from photosensitizers, which are prone to causing phototoxicity and skin allergies [73]. In recent years, inorganic sonosensitizers have shown great SDT potential due to their excellent physicochemical properties and versatility, and the main factor limiting their wide application is biosafety [74]. At present, most of the research on inorganic sonosensitizers focuses on the field of anti-tumor SDT [75].

**Fig. 3:**
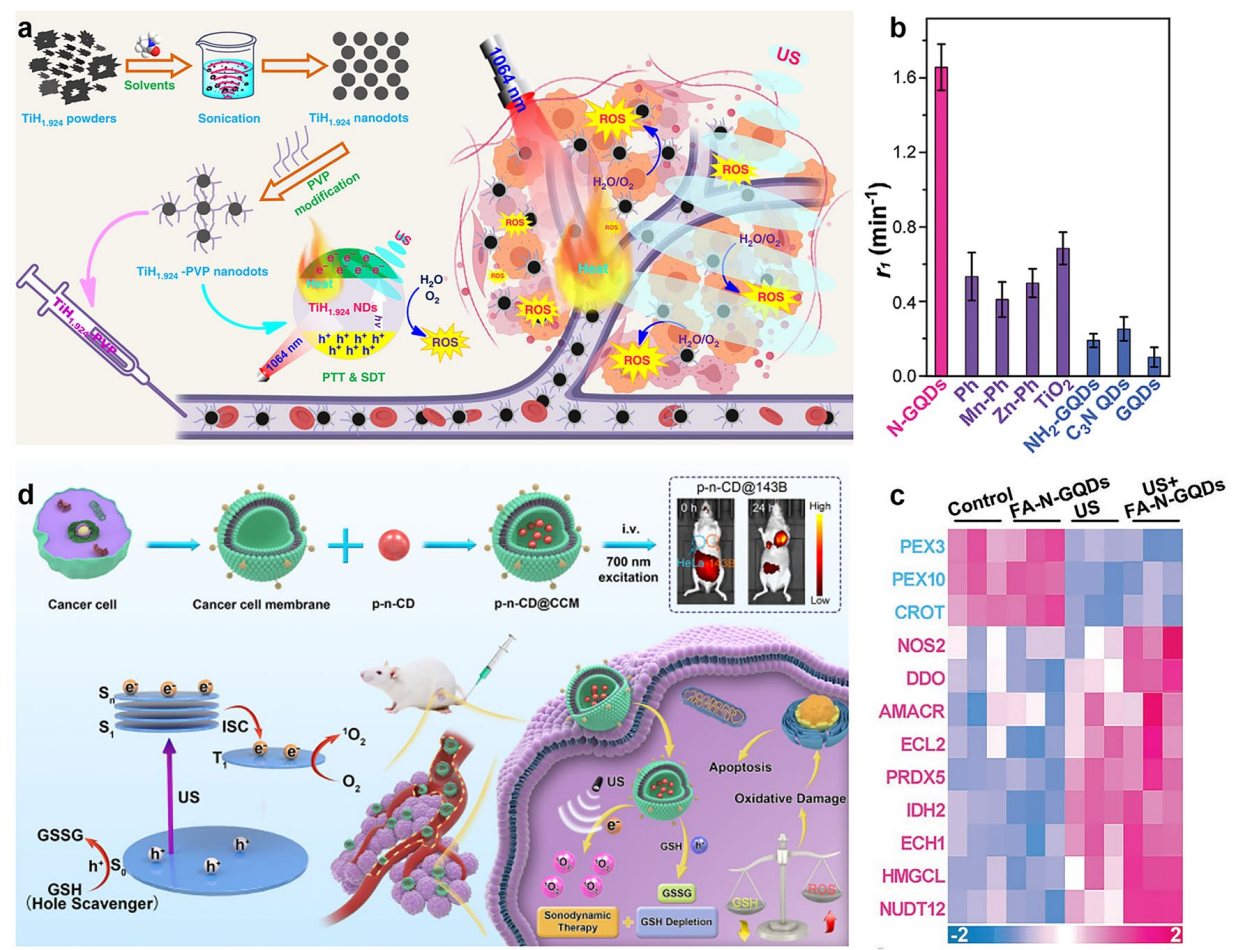
**a** Preparation of TiH_1.924_ nanodots used in the combination of SDT and PTT. Reproduced with permission [77]. Copyright 2020, Springer Nature. **b** Comparison of sonosensitization efficacy of N-GQDs with red with that of traditional sonosensitizers. **c** Oxidative stress-related proteins, indicating the oxidative stress response. Reproduced with permission [78]. Copyright 2021, John Wiley and Sons. **d** Preparation of p-n-CD@CCM and the application for SDT. Reproduced with permission [63]. Copyright 2022, Springer Nature

Titanium hydride finds extensive application in hydrogen storage, the production of metal sponges, and the manufacture of high-purity titanium. It exhibits responsiveness to external stimuli such as light, US, and microwaves, facilitating photocatalysis and sonocatalysis when its polyvalent titanium elements are stimulated [76]. Nevertheless, the utilization of nanoscale titanium hydride in the field of biomedicine remains unreported thus far. Liu et al. have demonstrated the efficient fabrication of nanoscale metal hydrides (TiH_1.924_) through the liquid phase exfoliation method, with the stripping efficiency contingent upon the compatibility between the stripping reagent and the surface energy of the hydride material [77]. Subsequent experimental results reveal that the obtained TiH_1.924_ nanodots could generate ROS under US exposure, indicative of a pronounced sonosensitization effect. Additionally, these TiH_1.924_ nanodots exhibit strong NIR absorption, and their mild photothermal effect can enhance tumor blood flow and elevate oxygen levels, thereby enabling photothermal-sonodynamic synergistic therapy (Fig. 3a). Furthermore, a significant portion of the ultra-small TiH_1.924_ nanodots can be eliminated from the body, significantly enhancing their biosafety and paving the way for extensive applications of metal hydride nanomaterials in biomedicine.

Graphene quantum dots provide new ideas and methods for the development of SDT owing to their unique physical and chemical properties, including high stability, non-toxicity, and excellent sonosensitivity [63]. The group of Ding and their collaborators for the first time demonstrated the excellent sonosensitive activity of N-doped graphene quantum dots (N-GQDs) [78]. The ROS generation efficiency of N-GQDs under US exposure is 3 to 5 times higher than that of traditional sonosensitizers (Fig. 3b). This study is the first to clarify the sonochemical mechanism of N-doped carbon nanostructures. Both experimental and theoretical results indicate that pyrrole N and pyridine N in N-GQDs serve as the reaction sites for the sonochemical process. The clarification of the above mechanism has reference significance for the further structural design of high-performance carbon-based sonosensitizer. In addition, owing to the stability of pyrrole N and pyridine N within the graphene quantum dot lattice, N-GQDs retain exceptional sonosensitive activity even after tumor-targeting modification (obtaining FA-N-GQDs). Additional tests involving oxidative stress-associated proteins revealed that the abundant ROS generated by FA-N-GQDs under US irradiation could trigger the oxidative stress response in tumor cells with high p53 protein expression via the PEX pathway, ultimately enhancing tumor cell apoptosis through the p53 protein (Fig. 3c). The results of cell experiment also showed that the cell killing efficiency of FA-N-GQDs exposed to the US was greater than 95%. In addition, the results of the treatment of a mouse subcutaneous tumor model demonstrated that the targeted modification of FA-N-GQDs could rapidly and stably accumulate in tumor tissue, and the tumor volume was reduced by more than 95% after two US irradiations within 14 days. At the same time, due to the excellent stability and biosafety of FA-N-GQDs, no significant toxic side effects were found during the treatment.

Furthermore, Pan et al. proposed the concept of "phosphorescent sonosensitized agent" for the first time, designed and prepared a new type of NIR phosphorescent carbon point, and applied it to the precision SDT tumor treatment mediated by NIR imaging [63]. By using a one-step microwave synthesis strategy to regulate the conductive type of carbon dots, the homogeneous *p*–*n* junction carbon dots prepared not only have NIR phosphorescence characteristics but also have enhanced sonodynamic activity (Fig. 3d). The excellent SDT is reflected in (1) *p*–*n* junction can effectively suppress the recombination of electron–hole pairs; (2) the excited long-lived triplet can produce singlet oxygen efficiently; (3) stimulated holes can efficiently consume GSH in the TME. Finally, complete eradication of osteosarcoma in tumor-bearing mouse models was achieved by a single intravenous injection and single US after encapsulation of NIR phosphor carbon points with homologous targeting cancer cell membranes. This work not only opens up a new way for the long-life triplet of phosphor materials to be used in NIR image-mediated SDT, but also provides a new idea for the application of quantum dot phosphor materials in near-infrared light-emitting devices.

## Nanosonosensitizers Enhancing the Efficacy of SDT

While the advantages of SDT arouse wide concern, its therapeutic effectiveness is influenced by a number of factors, such as the inherent structure of the sonosensitizer, as well as the high GSH level and hypoxic environment in the tumor microenvironment. With the blossom of nanotechnology, nanomaterials provide new prospects and development directions for enhancing the treatment of cancer [41, 79]. In view of the above issues, various construction strategies utilizing nanomaterials are implemented to boost the therapeutic outcomes. Based on these strategies, nanomaterials with different characteristics are divided into four categories: enhancing tumor accumulation, imaging guidance, reducing endogenous antioxidant GSH, and overcoming hypoxia (Table 1).

**Table 1:** Summary of various strategies for nanosonosensitizers with diverse characteristics

Category	Nanosonosensitizer	Strategy	Efficacy	References
Enhancing tumor accumulation	Chlorophyll loaded into nanoparticle	EPR effect via nanodelivery systems	Better therapeutic effects than chlorophyll alone	[83]
	FA-MnPs	Modify FA on nanoparticles	Good ultrasonic penetration response and anti-tumor immunity	[86]
	CCP@HP@M	Modify homologous cell membranes on the nanocarriers	Enhance SDT by synergistic ROS enhancement and autophagy block	[90]
	SPNC1	Reduce the size of the nanoparticles	Enhancing the sonodynamic treatment effect on large tumor models	[91]
Improving the safety with image-guiding therapy	prodrug NPs	Fluorescence image-guiding SDT	Enhanced fluorescence at the tumor site conducive to tumor edge recognition	[93]
	P-DOA NPs	Photoacoustic image-guiding SDT	Real-time photoacoustic imaging facilitates therapy	[95]
	PEGylated MnWOX	Multimodal image-guiding SDT	Highly efficient tumor-guiding ability	[96]
Reducing endogenous antioxidant GSH	MoOX-PEG	Metal oxides directly react with GSH	GSH-enhanced SDT with a good killing effect	[99]
	HCIr	Consume GSH by accelerating the Ir(IV)-Ir(III) transition	Enhancing the death of iron-like death cancer cells	[100]
Overcoming hypoxia	DOX/Ce6@ZIF-8@PDA	Oxygen storage capacity of MOFs alleviates tumor hypoxia	O_2_ supplementation contributes to US-induced ROS production	[108]
	MA-CPPNDs@O_2_	PFH provides oxygen	Enhance the tumor-killing effect of SDT	[109]
	HMV	Catalyze H_2_O_2_ in TME to generate oxygen	Improving the efficacy of SDT	[105]
	H-Ti_3_C_2_-PEG NSs	Accelerate blood flow	Tumor growth was completely inhibited	[110]
	TiO_2_/C	Type I sonosensitizers with less oxygen dependence	Effective treatment of hypoxic solid tumor	[113]
	PALF	Inhibiting cell aerobic respiration and reducing cell oxygen consumption	Enhances SDT effect and inhibits tumor proliferation and metastasis	[114]

### Enhancing Tumor Accumulation

Sonosensitizer is an indispensable component of SDT. However, the clinical application of SDT has been hindered by the scarcity of effective sonosensitizers. Most traditional photosensitizers can be used as sonosensitizers, but due to their hydrophobic essential structure, they are easy to accumulate in the physiological environment, which seriously affects their bioavailability, biocompatibility, and targeting ability, resulting in insufficient tumor accumulation and poor therapeutic effect [80]. Nanocarriers can help deliver sonosensitizers more efficiently at target tumor sites while increasing their sonodynamic effects by enhancing permeability and retention (EPR) effects [81, 82]. In addition, the nanoparticles provide nucleation sites for the formation of bubbles, thereby improving the efficiency of SDT. The development of novel nanosonosensitizer with good biocompatibility, high bioavailability, high specificity, high sonosensitive efficiency, and optimized US regulations is key to guiding the successful clinical conversion of SDT. Barbara Stella et al. added unmodified plant-extracted chlorophyll to nanocarriers with different compositions and structures to obtain differ aqueous formulations [83]. The effects of different formulations on the sonodynamic effects of chlorophyll were evaluated by incubation with human prostate cancer cells (PC-3) and spheroids (DU-145). Compared with chlorophyll alone, SDT using different nanocarriers showed better therapeutic effects, which further demonstrates the critical role of nanodelivery systems in SDT.

**Fig. 4:**
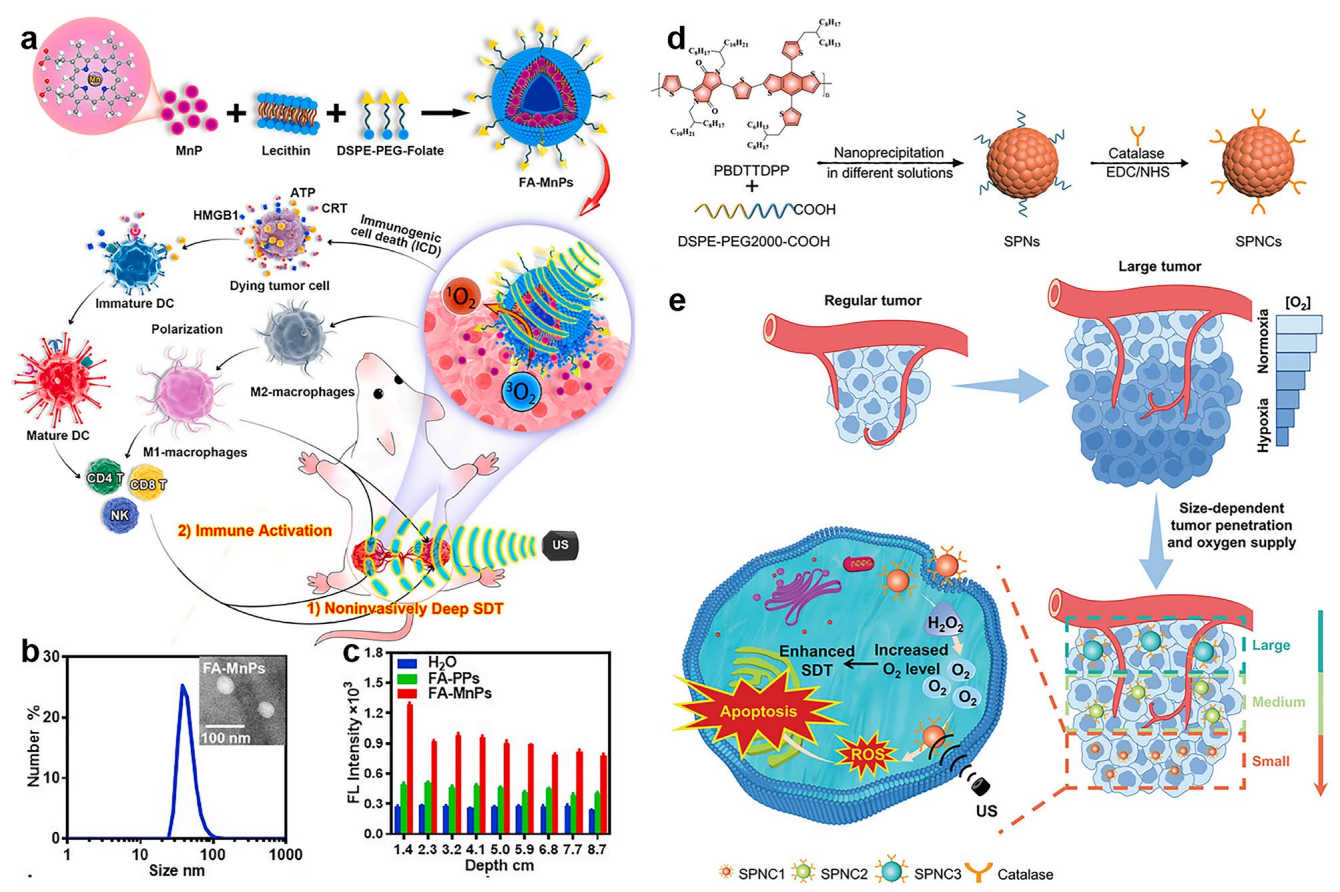
**a** FA-MnPs-mediated deep penetration of SDT and immunoactivation for tumor suppression. **b** DLS profile and TEM image of FA-MnPs. **c** ROS produced by ultrasonic activation of samples at different depths of tissue simulation [86]. Copyright 2021, Elsevier. **d** Construction of SPNCs with different sizes. **e** Size-dependent penetration ability for enhanced SDT of SPNC1 in the large tumor model [91]. Copyright 2022, John Wiley and Sons

In addition to passive targeting, small molecules identified by overexpressed receptors at tumor sites can be more conducive to tumor targeting [84, 85]. For example, considering that folic acid (FA) receptors have been reported to be overexpressed in a variety of cancers, Cai et al. have developed a multifunctional nanosonosensitization system (FA-MnPs), which is mainly formed by FA-modified liposomes coated with manganese-protoporphyrin (MnP) complexes to increase the accumulation of MnP in tumor cells [86]. Based on FA's tumor-targeting ability, the accumulation and metastasis of FA-MnPs in tumor cells significantly increased (Fig. 4a). The results show that FA-MnPs present a uniform spherical with a diameter of hydration radius of about 50 nm (Fig. 4b), which facilitates targeting and penetrating tumor tissue, avoiding clearance by the reticuloendothelial system. FA-MnPs also have good ultrasonic penetration response in deep tumor tissues, producing a large number of ROS at penetration depth up to 8 cm in a mouse model of triple-negative breast cancer (TNBC) (Fig. 4c). In addition, FA-MnPs-mediated SDT induces immunogenic cell death (ICD) activation of dendritic cells, T lymphocytes, and natural killer cells, and further repolarization of immunosuppressed M2 macrophages into anti-tumor M1 macrophages, thus triggering anti-tumor immunity and inhibiting tumor growth. This study provides a good strategy for non-invasive immunogenic SDT treatment of deep tumors and metastatic tumors.

In addition, due to the homologous targeting properties of cancer cell membranes, modification of nanomaterials with homologous tumor cells is also helpful to achieve targeted delivery [87–89]. The team of Zhang synthesized a cascade nanoreactor, which could enhance SDT for colon cancer by synergistic ROS enhancement and autophagy block [90]. Firstly, the autophagy inhibitor chloroquine and the sonosensitizer Ce6 were loaded into a hollow polydopamine nanocore, which was predoped with platinum nanocases (CCP@HP). Secondly, homologous cell membranes were used to carry out surface functionalization modifications on the nanocarriers (CCP@HP@M), so as to help the nanocarriers to better locate the tumor site and achieve a more significant therapeutic effect. Polydopamine nanocarriers (HP) have superoxide dismutase activity, which can convert O_2_^−^ to O_2_ and H_2_O_2_, and platinum nanocases further catalyze H_2_O_2_ to produce toxic ·OH and O_2_. Under US irradiation, CCP@HP@M can effectively relieve the hypoxic state of the tumor, enhance the generation of ROS, inhibit the protective autophagy pathway, and induce apoptosis and iron death. By regulating ROS and autophagy sensitizing SDT, the cascade nanoreactor provides a new idea for the precise treatment of deep tumors.

SDT has broad application prospects in the treatment of deep tumors or large tumor models due to its deep-tissue penetration ability, but its therapeutic effect is often limited by the tumor penetration ability of the sonosensitizer and the hypoxic TME. To solve these problems, the group of Jiang reported a series of semiconducting polymer nanoparticles (SPNCs) with different particle sizes and studied the SDT of SPNCs in large tumor model [91]. PBDTTDPP, a semiconductor conjugated polymer with sonodynamic properties, and DSPE-PEG2000-COOH, an amphiphilic polymer, were fabricated into nanomaterials with different particle sizes by nanoprecipitation method, and catalase was modified on their surfaces to obtain three nanomaterials with different particle sizes, SPNC1(35 nm), SPNC2 (84 nm), and SPNC3 (134 nm) (Fig. 4d). All of the three SPNCs showed excellent sonodynamic performance, similar cell uptake capacity, cytotoxicity, and tumor enrichment capacity. However, thanks to its smallest particle size, SPNC1 demonstrated the strongest 3D tumor cell and solid tumor tissue penetration in both 3D cell and animal studies. The catalase can effectively improve the tumor hypoxic microenvironment, thus enhancing the sonodynamic treatment effect on large tumor models (Fig. 4e).

### Improving the Safety with Image-Guiding Therapy

As a visible tool, imaging technology monitors the distribution of sonosensitizers in the body in real time. Thus, the image-guided treatment model can effectively improve the efficiency treatment and reduce damage to surrounding normal tissue [92]. The nanomaterials integrate therapy and imaging into a single platform to achieve image-guided SDT, thus achieving the ultimate goal of tumor eradication.

#### Fluorescence Image-Guiding SDT

**Fig. 5:**
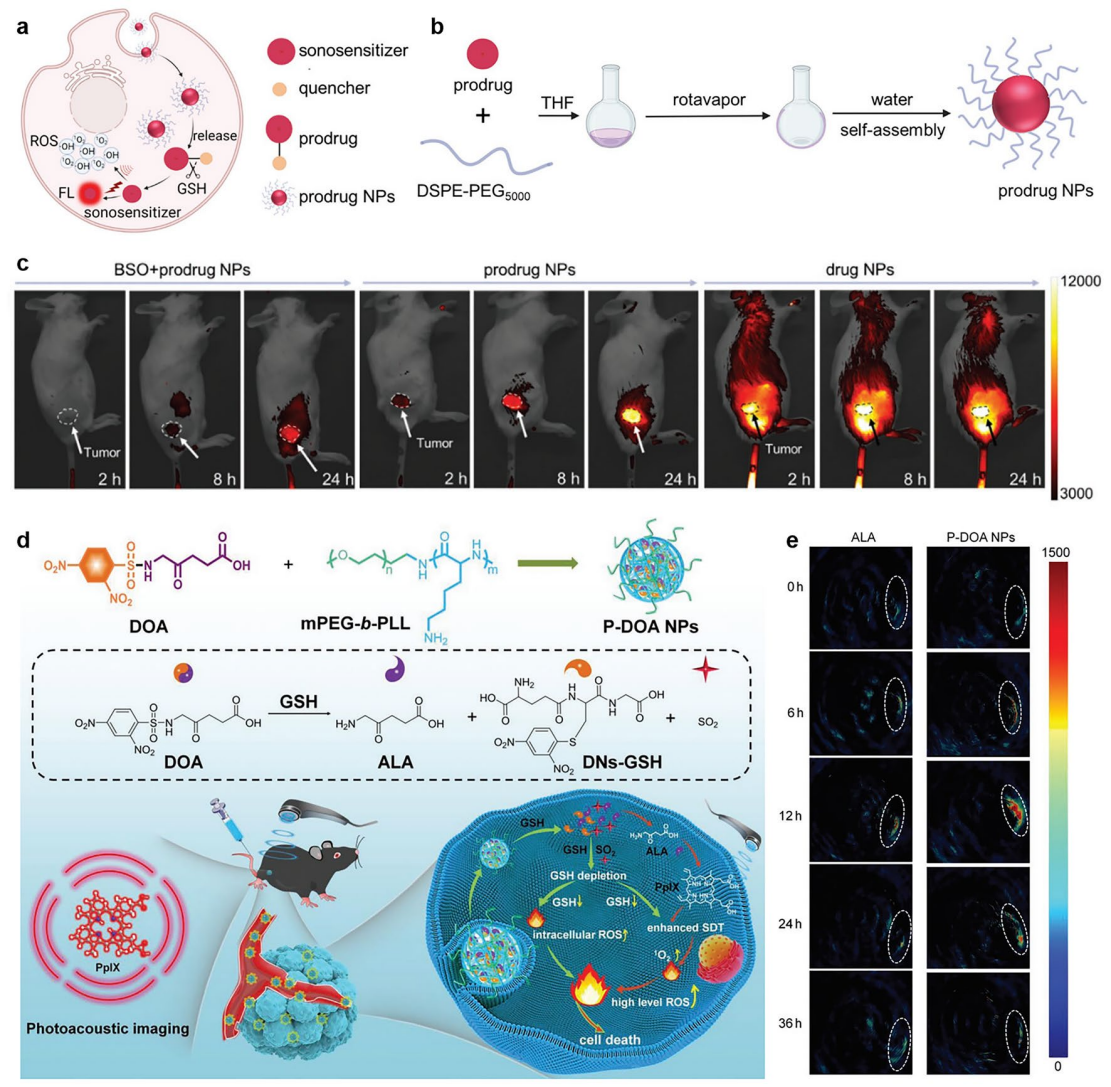
**a** Schematic diagram of prodrug NPs being activated simultaneously turning on fluorescence emission and ROS generation. **b** Preparation of prodrug NPs. **c** Fluorescence imaging of tumor-bearing mice injected with different samples [93]. Copyright 2023, John Wiley and Sons. **d** Preparation of P-DOA NPs and their application in enhancing SDT under the guidance of photoacoustic imaging. **e** Photoacoustic imaging of tumor sites in tumor-bearing mice [95]. Copyright 2022, John Wiley and Sons

An et al. designed a GSH-activated sonosensitizer prodrug by connecting the quenching group 2, 4-dinitrobenzenesulfonyl to tetrahydroxy porphyrin that can be selectively activated at tumor sites for fluorescence image-guided SDT (Fig. 5a) [93]. Nanoparticles were constructed using DSPE-PEG5000 to disperse the prodrug so that the prodrug could be used in vivo (Fig. 5b). Based on fluorescence imaging, mice injected with the prodrug showed enhanced fluorescence at the tumor site, conducive to tumor edge recognition, which was used to precisely guide US exposure during SDT (Fig. 5c). The US irradiation showed effective tumor growth inhibition with no observable side effects on normal organs, enabling accurate cancer treatment diagnosis. Besides, Wang and collaborators have developed a dual-sensitizer prodrug, pro-THPC, that could be used as both a photosensitizer and a sonosensitizer prodrug for precise anti-tumor combination therapy while minimizing skin phototoxicity [94]. Pro-THPC can be activated by GSH, releasing the double sensitizer THPC, while turning on fluorescence emission and the combined function of PDT and SDT. Pro-THPC is further formulated into nanoparticles (pro-THPC NPs) for water dispersion, enabling in vivo applications. In vivo fluorescence imaging showed that the ratio of tumors to normal tissue was significantly higher in the pro-THPC NPs group compared to the “always on” THPC NPs group. In addition, due to the strong correlation between ROS generation and fluorescence emission, the generation of the designed double sensitizer ROS is effectively limited to tumor tissue.

#### Photoacoustic Image-Guiding SDT

Compared with fluorescence imaging, photoacoustic imaging has significant superiorities in imaging depth, spatial resolution, and multi-scale imaging, so that it has a wider application prospect in image-guided therapy [94]. The group of Jiang developed two-in-one prodrug nanoparticles (P-DOA NPs) for photoacoustic imaging-guided SDT. Firstly, they synthesized a dual-prodrug molecule (DOA), which could release ALA and sulfide dioxide (SO_2_) for enhanced SDT [95]. Then P-DOA NPs were constructed based on the self-assembly of the obtained DOA and methoxyl poly(ethylene glycol)-*b*-poly (L-lysine) (mPEG-b-PLL) by forming dynamic Schiff base bonds, electrostatic interactions, and hydrophobic interactions (Fig. 5d). Upon responding to GSH in tumor cells, P-DOA NPs could release both SO_2_ and ALA, which is then metabolized into PpIX for photoacoustic imaging-guided SDT. Moreover, the reaction of DOA in P-DOA NPs with intracellular GSH reduced the content of GSH and thus reduced the consumption of ROS. At the same time, the release SO_2_ can significantly increase the intracellular ROS content. Both of them ultimately enhance SDT. Consequently, P-DOA NPs exhibited notable inhibition of melanoma and squamous cell carcinoma xenograft growth in mouse models, facilitated by real-time photoacoustic imaging (Fig. 5e). Hence, this innovative 2-in-1 nanoprodrug holds promise for effective skin cancer treatment through SDT.

#### Multimodal Image-Guiding SDT

In addition, Liu et al. designed a novel sonosensitizer, ultra-small anoxic bimetallic oxide MnWOX nanoparticles, for multimodal imaging-guided enhancement of SDT anti-cancer [96]. The PEGylated MnWOX has good physiological stability and biocompatibility. This MnWOX-PEG nanoparticle exhibits highly efficient ^1^O_2_ and ·OH generation after triggered by US, since the anoxic structure of MnWOX acts as an electron trap site to prevent electron–hole recombination. Intriguingly, MnWOX-PEG can not only perform fluorescence imaging, but also magnetic resonance and computer-tomography imaging. Multi-modal imaging results confirm that MnWOX-PEG has a highly efficient tumor-guiding ability and can effectively destroy mouse tumors under US stimulation. After completion of treatment, MnWOX-PEG can be metabolized in mice without long-term toxicity.

### Reducing Endogenous Antioxidant GSH

Tumor cells metabolize rapidly and the tumor tissue does not have adequate blood supply, so the GSH content at the tumor site is often significantly higher than that found in normal tissue [97]. ROS exhibits a pivotal role in numerous cancer treatments; however, GSH within tumors clears ROS, leading to increased resistance of tumor cells to oxidative stress and reducing the effectiveness of cancer treatments. Therefore, reducing biosynthetic antioxidants such as GSH is one of the effective strategies to improve the therapeutic effect.

Since some metal oxides have certain oxidation properties, they can directly react with GSH [98]. For example, molybdenum oxide (MoO_X_), as an important semiconductor material, has received extensive attention in many fields due to its unique structure and outstanding optical properties. The group of Cheng constructed anoxic MoO_X_ nanomaterials as novel nanosonosensitizers for ultrasonic enhancement of oxidative stress [99]. MoO_X_-PEG modified with polyethylene glycol (PEG) demonstrates efficient ROS generation ability under US irradiation, increased oxidative stress in tumors, induced ICD, and effectively inhibited tumor growth. At the same time, MoO_X_-PEG nanomaterials disrupted redox homeostasis by consuming endogenous GSH, weakened tumor antioxidant capacity, and showed GSH-enhanced SDT with a good killing effect in vitro and in vivo, which was mainly due to electron capture to delay the increase of electron holes to the reconstituted anoxic structure.

In addition, Hu's group developed an iron death inducer, human serum albumin-iridium oxide (HSA-Ce6-IrO_2_, HCIr) nanocluster of conjugated Ce6, and used it to achieve iron-like death of cancer cells triggered by SDT [100]. Ce6 and IrO_2_ in the HCIr nanoclusters are acoustically sensitive and can efficiently generate ^1^O_2_ under US stimulation, promoting the accumulation of lipid peroxides (LPO), and then inducing iron death. At the same time, HCIr can also consume GSH by accelerating the Ir(IV)-Ir(III) transition, thereby inhibiting GSH peroxidase 4 (GPX4) activity and enhancing the efficacy of iron death. Both in vitro and in vivo results displayed that HCIr can significantly reduce the intracellular GSH content, thereby enhancing the death of iron-like death cancer cells induced by SDT.

**Fig. 6:**
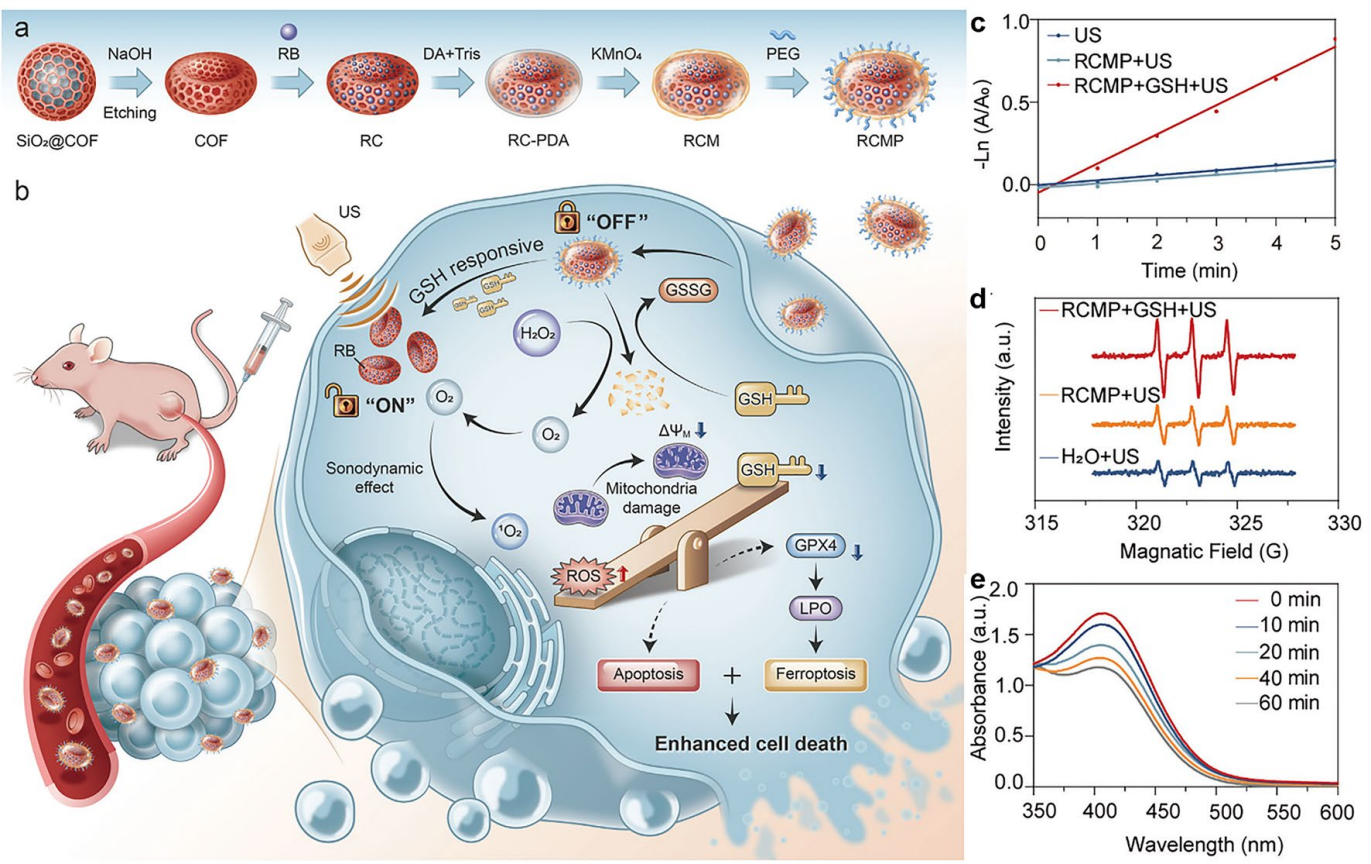
Preparation of RCMP for the enhanced anti-cancer therapy. **a** Construction of the nanomaterials RCMP. **b** Cancer cell death is synergistically enhanced by enhanced oxygen release and GSH consumption. **c** ROS generation using DPBF as the probe. **d** ROS generation using 2,2,6,6-tetramethylpiperidine (TEMP) as probe. **e** GSH depletion by RCMP. Reproduced with permission [102]. Copyright 2023, John Wiley and Sons

Covalent organic framework (COFs) is widely used as a porous material in many biomedical fields. Nanoscale COFs have controllable morphology, which can effectively improve their physicochemical properties [101]. Therefore, the controlled synthesis of nanoscale COFs with unique morphology and high crystallinity is of great significance for expanding their application. Zhou and the coworkers developed COF nanobowls with distinctive morphology, which could effectively load small-molecule sonosensitizer RB and then seal MnO_x_ by a dopamine-mediated redox reaction without affecting the high crystallinity of the COFs [102]. Finally, the target nanosonosensitizer (RB@COFs-MnO_x_-PEG, named RCMP) was obtained via modification with polyethylene glycol for enhanced SDT (Fig. 6a, b). The experiment results have shown that a high concentration of GSH in tumor cells can cause MnO_x_ collapse, thereby restoring the sonosensitive activity of RB to achieve the sonodynamic process in cancer cells (Fig. 6c, d). In addition, the catalytic action of MnO_x_ also promoted the release of intracellular oxygen and the consumption of GSH (Fig. 6e), which improved the effect of SDT. The enhancement of ROS combined with the consumption of GSH significantly enhanced the therapeutic effect. In vivo evaluation also confirmed that the bowl-like morphology enables COF nanosensitizers a special enhancement effect on tumor accumulation and retention.

### Overcoming Hypoxia

The proliferation of cancer cells is a highly energy-intensive process, but the blood circulation can not meet the tumor tissue's demand for O_2_ [103, 104]. Due to the imbalance of oxygen supply and consumption, 50% to 60% of malignant solid tumors result in hypoxia, which is a major factor in tumor metastasis and angiogenesis, and can also lead to a decrease in ROS produced by O_2_-dependent PDT, SDT, and other therapies, greatly reducing the therapeutic effect [105]. Therefore, regulating the hypoxic microenvironment of the tumor can help to inhibit the therapeutic resistance of the tumor and improve the therapeutic effect. In recent years, various studies have reported that the delivery of O_2_ to tumors by blood metabolites (such as perfluorocarbons) or the use of catalysts to produce O_2_ in situ can improve cancer treatment efficacy to some extent by overcoming tumor hypoxia.

#### Relieving Oxygen Concentration

Since the production of type II ROS is heavily dependent on the concentration of oxygen, the anoxic environment hinders the effect of SDT [106]. Therefore, there is an urgent need to develop sonosensitizers with self-supporting O_2_ capacity to increase ROS levels and thus improve the therapeutic effect of SDT.

**Fig. 7:**
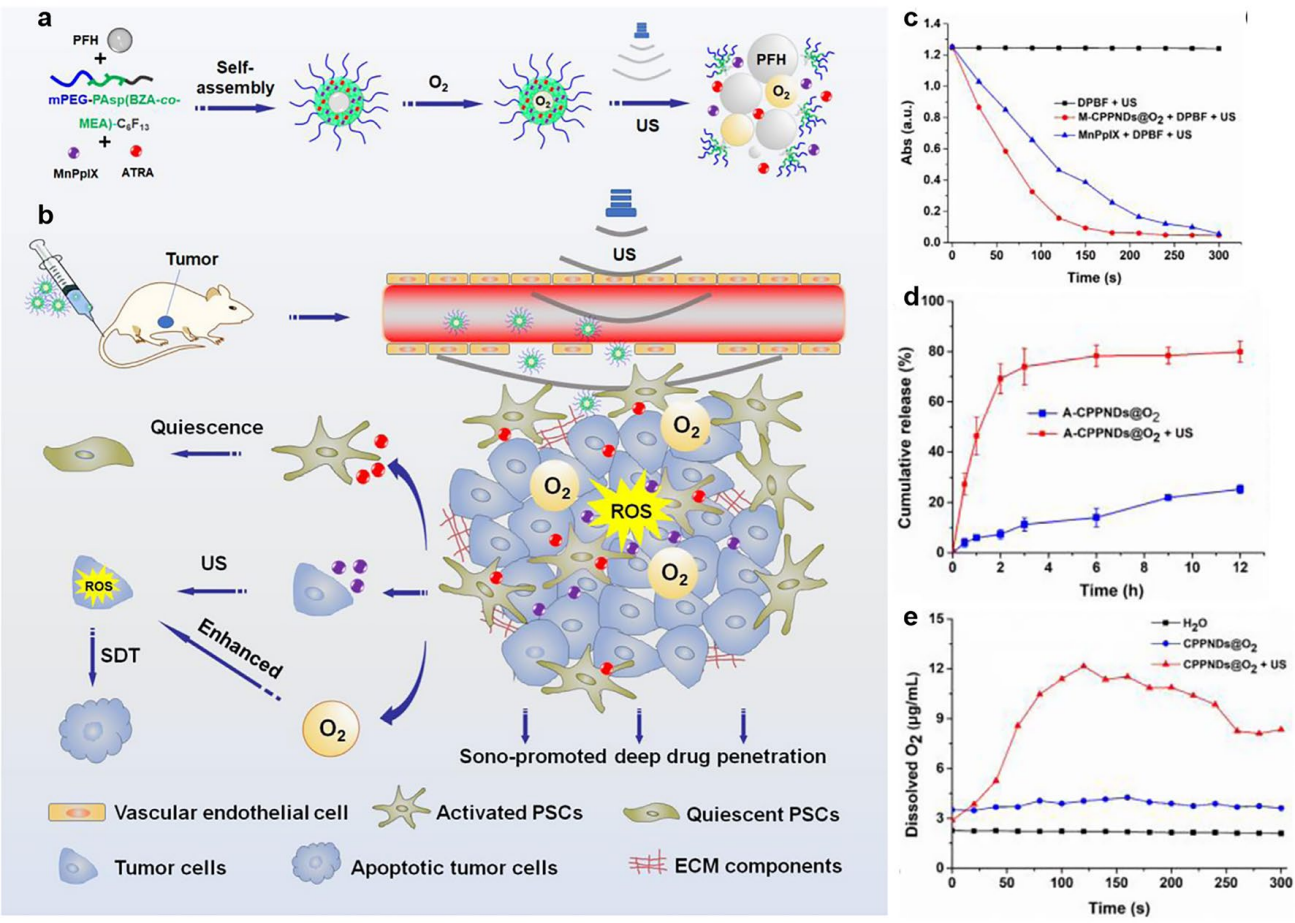
Polymeric PFH nanodroplets for the enhanced SDT. **a** Construction of MA-CPPNDs@O_2_ and releasing O_2_ under US irradiation. **b** MA-CPPNDs@O_2_ promoted the deep penetration of drugs into pancreatic cancer tissues for better treatment. **c** ROS generation of M-CPPNDs@O_2_ using DPBF as the probe. **d** ATRA and **e** O_2_ release of MA-CPPNDs@O_2_ [109]. Copyright 2023, Elsevier

The most direct way to alleviate hypoxia is to deliver oxygen to the TME [107]. Gu et al. constructed core–shell nanostructures of DOX/Ce6@ZIF-8@PDA to destroy tumor hypoxia and enhance SDT [108]. Notably, the oxygen storage capacity of MOFs alleviates tumor hypoxia, making tumors sensitive to SDT and chemotherapy. The oxygen storage capacity of MOFs could alleviate tumor hypoxia and make tumors more sensitive to SDT. Under US exposure, a large amount of oxygen can alleviate tumor hypoxia and make Ce6 rapidly produce rich cytotoxic ^1^O_2_. The in vivo and in vitro studies have verified that the O_2_ supplementation contributes to US-induced ROS production. For specific load release and local oxygenation in tumors, Shuai’s group developed an US-responsive polymer perfluorohexane (PFH) nanodroplet (MA-CPPNDs@O_2_), which promoted the deep penetration of drugs into pancreatic cancer tissues by combining exogenous ultrasonic stimulation and endogenous ECM regulation, and was used for efficient SDT of pancreatic cancer [109]. The nanodroplet is formed by self-assembly of the fluoroalkane-modified polymer mPEG-PAsp (Beza-CO-MEA)-C6F13 with all-trans retinoic acid (ATRA), manganese porphyrin (MnPpIX), and oxygen-rich PFH (Fig. 7a). Under US irradiation, the nanodroplets vaporize rapidly and generate cavitation force, promoting the rapid release of loaded drugs from the nanodroplets and deep penetration in pancreatic cancer tissue. At this time, well-permeated ATRA effectively inhibited the secretion of ECM protein components by pancreatic stellate cells (PSCs), reduced the density of the pancreatic cancer matrix, and formed a non-dense microenvironment beneficial to drug diffusion (Fig. 7b). At the same time, MnPpIX activated by US produced a large number of reactive oxygen species and exerted an SDT effect. Using PFH as the transport medium of O_2_, nanodroplets can also effectively alleviate the hypoxia of tumor tissue and enhance the tumor-killing effect of SDT. The in vitro ROS generation was tested using DPBF as the probe. The ROS generation of M-CPPNDs@O_2_ + DPBF + US was higher than that of MnPpIX + DPBF + US due to the delivered O_2_ by M-CPPNDs@O_2_ (Fig. 7c). Besides, ATRA and O_2_ could be effectively released by M-CPPNDs@O_2_ under US (Fig. 7d, e). The nanodroplets effectively inhibited the growth of pancreatic cancer tumors.

In addition, catalyzing H_2_O_2_ in TME to generate oxygen is also one of the effective strategies to increase oxygen concentration. Wang et al. designed a hypoxic-responsive nanovesicle (HMV) with increased oxygen concentration in situ [105]. The nanovesicles consist of densely packed manganese ferrite nanoparticles (MFNs) encased in a hypoxia-responsive amphiphilic polymer film while loaded with δ-aminolevulinic acid (ALA) in a hollow cavity. After intravenous injection, ALA-loaded MFN vesicles (ALA-HMVs) remained stable under normal physiological conditions, effectively preventing non-specific leakage of ALA and passively accumulating in tumors through EPR effects. After arriving at the tumor site, the amphiphilic polymer transforms into hydrophilic after responsive to hypoxia, thus releasing ALA by specific dissociation, and ALS transforms into PpIX under US stimulation to induce ROS generation. At the same time, the dissociated ALA-hMVs can also release MFNs as a highly efficient catalyst to generate O_2_ from overexpressed H_2_O_2_ in TME and overcome the hypoxic TME, thus improving the efficacy of SDT. After treatment, dissociated MFNs can be excreted by renal clearance, reducing long-term toxicity, which has the potential to aid in the progression of clinical translation. In vitro and in vivo experiments have shown that SDT mediated by HMVs can effectively inhibit tumors, suggesting that this unique nanoplatform can generate enough ROS in deep hypoxic tumors that are difficult to be reached by PDT to achieve effective SDT.

The utilization of stimulation to accelerate blood flow can also improve oxygen levels. In order to solve the problem of reduced ROS caused by hypoxia, Cheng and colleagues synthesized an MXene-based sonosensitizer (H-Ti_3_C_2_-PEG NSs) through a two-step method, which extended blood circulation through a mild photothermal effect and effectively improved oxygen supply to enhance SDT [110]. Moreover, under the exposure of 1064-nm light and US together on H-Ti_3_C_2_-PEG NSs, the tumor growth was completely inhibited, and the survival of mice was greatly prolonged.

#### Reducing Oxygen Consumption

Although the strategy improves the efficiency of SDT to some extent by increasing the oxygen concentration within the tumor, these methods have serious side effects, such as safety concerns, promoting cancer cell proliferation and metastasis, and spatiotemporal controllability, which affect their practical application. Another way to overcome the limitations of anaerobic SDT methods is to develop sonosensitizers that are less dependent on O_2_.

**Fig. 8:**
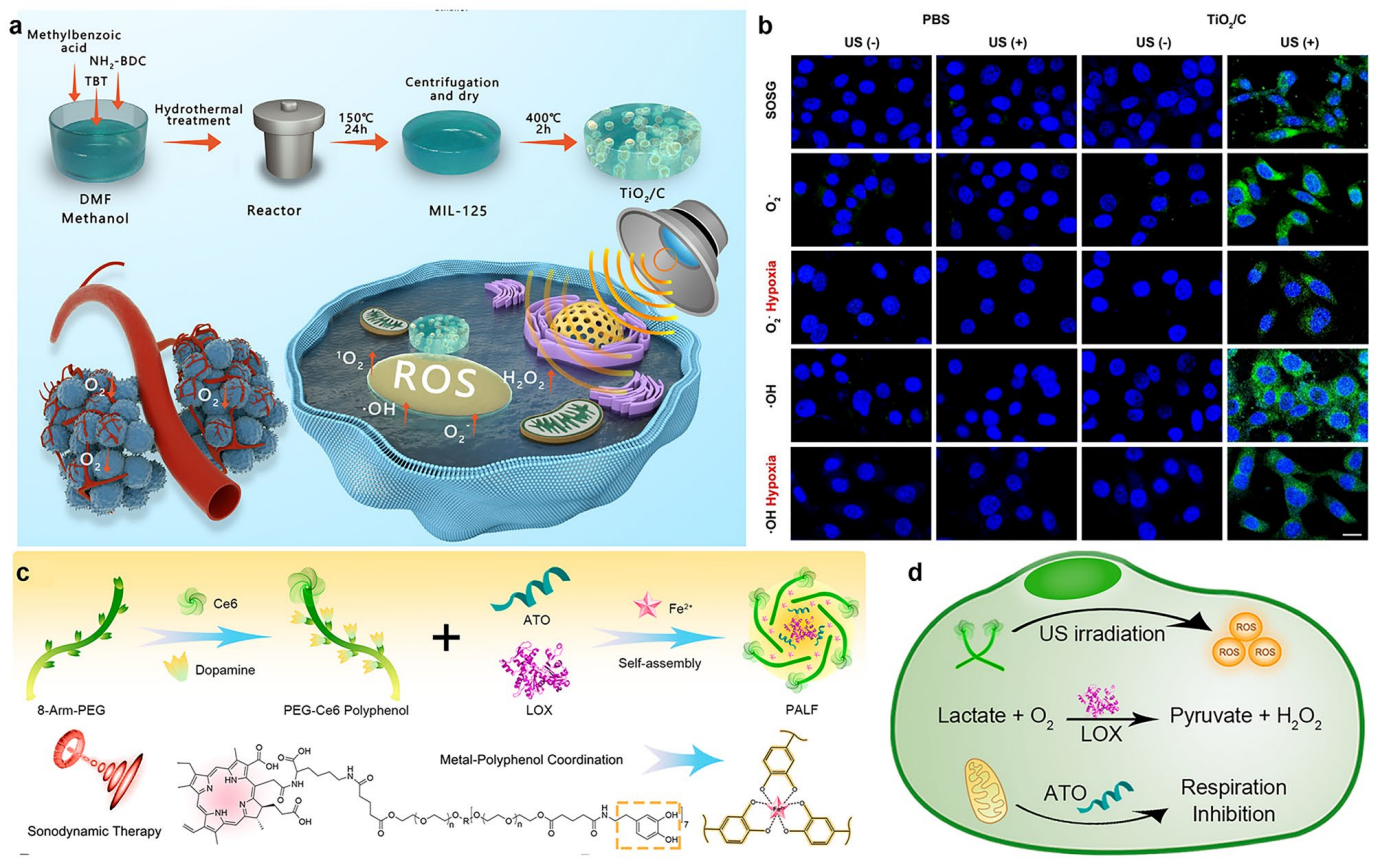
**a** Synthesis of TiO_2_/C and used for SDT. **b** Production of superoxide anions and hydroxyl radicals in cells with normal and hypoxic oxygen concentrations [113]. Copyright 2021, Elsevier. **c** Construction of PALF. **d** Major reaction of PALF at cellular level [114]. Copyright 2021, American Chemical Society

Type I sonosensitizers have been shown to have less oxygen dependence than traditional Type II sonosensitizers [111, 112]. The group of Huang reported a sheet carbon-embedded TiO_2_ nanocomposite derived from a metal–organic framework (MOF) structure, which was found that it was hypoxic resistant and stable to repeated ultrasonic irradiation, leading to the production of abundant TiO_2_/C-mediated ROS, so as to achieve effective type I SDT (Fig. 8a) [113]. The potential application of TiO_2_/C in SDT with hypoxic tumor cells was confirmed by the evaluation of ROS at normal oxygen concentration and cell level with hypoxia (Fig. 8b). Importantly, under repeated exposure to US, the nanocomposite persistently produced ROS, inducing tumor cell apoptosis through SDT-induced DNA damage. The TiO_2_/C nanocomposite has good biocompatibility and no obvious toxicity. In short, these results underscore that TiO_2_/C is a valuable nanocomposite that can facilitate repetitive type I SDT, rendering it a promising therapeutic instrument for addressing hypoxic solid tumors.

In addition, it can also effectively relieve the hypoxia at the tumor site by inhibiting cell aerobic respiration and reducing cell oxygen consumption. Dai et al. synthesized a metal-phenol network-based nanocomplex (named PALF) crosslinked with Fe^2+^ ions via an artificial polyphenol derivative (PEG-Ce6 polyphenol) (Fig. 8c) [114]. Lactate oxidase (LOX) and mitochondrial respiratory inhibitor atovaquone (ATO) were simultaneously introduced for immunosuppressive TME remodeling and SDT. Among them, the conjugated polyphenol derivatives of Ce6 can cause the production of tumor-lethal ROS under ultrasonic irradiation. It is particularly noteworthy that LOX is the catalyst of intracellular lactic acid exhaustion, while ATO leads to mitochondrial dysfunction, reducing oxygen consumption effectively alleviates tumor hypoxia, enhances SDT effect, and inhibits tumor proliferation and metastasis (Fig. 8d).

## Application of Sonodynamic Therapy in Orthotopic Tumor

Due to the poor tissue penetration ability of light, PDT is limited to the treatment of only superficial or bladder cancer [115, 116], while SDT utilizes the penetration performance of US to treat larger or deeper tumors, so as to achieve accurate treatment [63, 117, 118]. This is of great significance for deep tumors that are difficult to reach by traditional treatment, even orthotopic pancreatic cancer, glioma, etc. [119]. Moreover, the combination of SDT with other therapies can further improve the therapeutic effect as well as the tumor targeting and safety of the therapy.

### Pancreatic Cancer

Pancreatic cancer is one of the deadliest cancers, with very low survival rates and little improvement in recent decades [120–122]. Owing that the complex TME affects the efficacy of various therapies, effective treatment of pancreatic cancer remains a major challenge [123]. Thus, there is an urgent need to explore new treatments or more effective strategies. SDT shows potential advantages in the treatment of many types of tumors, especially for deep tumors such as pancreatic cancer [31, 50].

#### SDT

Because the generated ROS have a short lifetime and limited diffusion range [115], a large amount of evidence suggests that nanosensitizers close to DNA are more likely to induce oxidative damage and thus achieve better therapeutic effects. Ultra-small MOFs have good intrinsic nuclear targeting and can effectively produce ROS to promote SDT tumor therapy. For example, Huang et al. constructed a ultra-small Ti-tetrakis(4-carboxyphenyl)porphyrin (TCPP) metal–organic framework (MOF) as sonosensitizer, which can target nucleus, realizing the treatment of orthotopic pancreatic carcinoma [124]. The Ti-TCPP MOF can not only target the nucleus but also produce ROS under hypoxic conditions to promote the efficiency of SDT.

To enhance the retention of the sonosensitizer at orthotopic pancreatic cancer, Wang et al. developed a sonosensitizers-free sonocatalytic nanomissile, which was consisted of poly lactic-co-glycolic acid (PLGA) nanoparticles loading L-Arginine (LA) molecules and aptamer XQ2d [125]. LA molecules with adsorbed CO_2_ could release CO_2_ bubbles in response to the acidic TME and local US. And the CO_2_ bubbles enhanced the inertial cavitation triggered by US, further split H_2_O, and activate dissolved O_2_ to generate OH and ^1^O_2_, respectively, to achieve SDT. In addition, microjets and shock waves generated by inertial cavitation enhanced by CO_2_ bubbles could trigger powerful mechanical effects. These mechanical effects directly destroyed the blood vessels in the tumor, promoted thrombus aggregation, blocked the blood oxygen supply, changed the metabolic pathway of the tumor, and hence synergically starved the tumor cells. In particular, the existence of aptamer made the nanoparticles the ability to actively target the overexpressed transferrin receptor (CD71) in pancreatic cancer tissue, thereby enabling a large amount of sonosensitizers to accumulate in the tumor site and amplifying the treatment of orthotopic pancreatic cancer.

#### *CDT* + *SDT*

CDT as a novel anti-tumor therapy uses the TME to activate the nanodrug Fenton-like reaction to generate strong oxidizing hydroxyl radical for tumor-specific treatment [126–128]. This therapy converts hydrogen peroxide in the TME into highly toxic hydroxyl radical through a Fenton reaction or Fenton-like reaction without additional external effects, making CDT a new strategy to effectively regulate the TME to achieve tumor therapy [129]. Metal–organic frameworks (MOFs) have been found to be very effective as delivery carrier is used as drug delivery owing to their strong drug carrying capacity [108, 130]. In addition, since MOFs are composed of metal and organic linking groups, which metal ions such as Fe^2+^ and Cu^+^ can catalyze H_2_O_2_ in the TME through Fenton or Fenton-like reactions, generating ROS and resulting synergistic enhancement of therapeutic effect [107, 131].

**Fig. 9:**
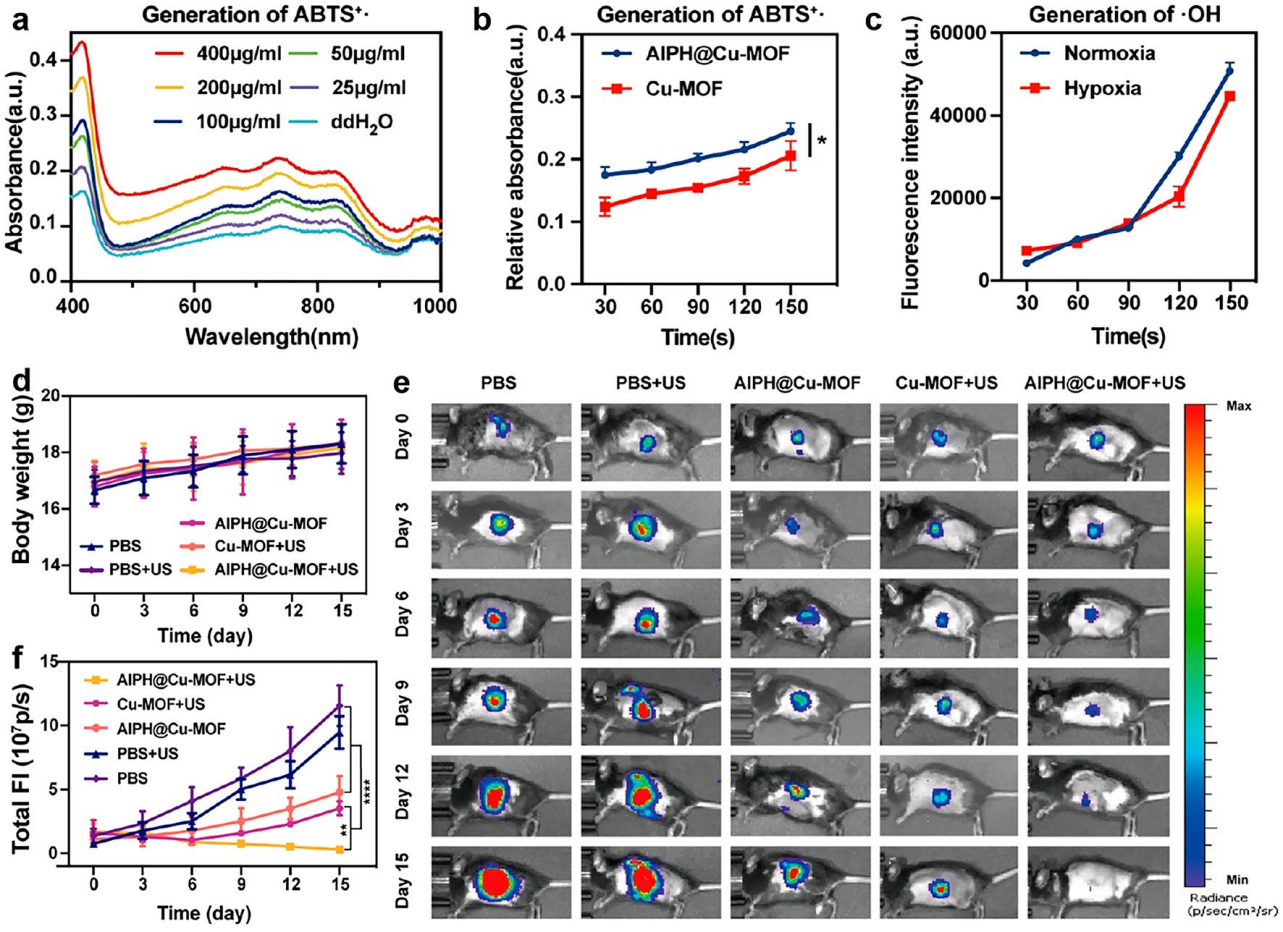
AIPH@Cu-MOF for the treatment of orthotopic pancreatic tumor. The generation of ABTS^+·^ **a** different AIPH@Cu-MOF concentrations and **b** with US irradiation. **c** ·OH generated of AIPH@Cu-MOF. **d** Body weight **e** bioluminescence imaging and **f** the fluorescence intensity of the Panc02-Luc orthotopic tumor-bearing mice over time [132]. Copyright 2021, American Chemical Society

For instance, Huang et al. developed AIPH@Cu-MOF nanoparticles (NPs) for the treatment of orthotopic pancreatic tumor by loading 2,2-azobis[2-(2-imidazolin-2-yl) propane] dihydrochloride (AIPH) into the hypoxia-responsive copper MOFs (Cu-MOF) [132]. In the hypoxic TME, the AIPH@Cu-MOF NPs released Cu and AIPH. AIPH produced cytotoxic alkyl radicals when stimulated by US (Fig. 9a, b), while GSH in the microenvironment reduced Cu^2+^ to Cu^+^, which could inhibit the consumption of ROS by GSH. Furthermore, Cu^+^ could effectively catalyze Fenton-like reactions in weakly acidic TME, resulting in significant ROS generation (Fig. 9c). To further verify the synergistic enhancement efficacy of AIPH@Cu-MOF NPs, an orthotopic pancreatic tumor model was established. Through the detection of body weight and tumor growth of mice (Fig. 9d-f), it was obviously found that the group of Cu-MOF preparation had significant tumor inhibition compared with the control group, indicating that CDT had a therapeutic effect in this pancreatic cancer model. Prominently, tumor growth inhibition was more obvious in the AIPH@Cu-MOF + US group than in the CuMOF + US group, suggesting that AIPH coupling and subsequent exposure to US significantly boosted the antitumor efficacy of these MOFs.

#### *SDT* + *Immunotherapy*

Pancreatic cancer tumor cells have a high likelihood of spreading and metastasizing. Thus, it is hard for chemotherapy and radiation to effectively treat them. Immunotherapy, mainly utilizing the immune system to specifically identify, attack, and destroy cancer cells, has become one of the main methods of cancer treatment [133]. Compared with other treatments, immunotherapy exhibits dual benefits, not merely restricting tumor growth and metastasis but also fostering a long-lasting immune memory that effectively safeguards against tumor recurrence [134, 135]. In preclinical models of cancer, SDT has exhibited the ability of stimulating the adaptive immune system [136]. The tumor cell fragments generated via the SDT process could be used as tumor antigens source and induce anti-tumor immune effects in the host [137].

For instance, Callan et al. developed a kind of nanomicrobubble that could induce necrosis and apoptosis for SDT [138]. Under ultrasonic exposure, nanomicrobubble could mediate cell membrane breakdown, effectively promote the release of molecular pattern molecules related to biological activity damage, and significantly improve anti-tumor immunity through the maturation of dendritic cells and the activation of CD8 + cytotoxic *T* cells. In addition, the combined application of nanomicrobubble and immune checkpoint blockers can completely regress the primary tumor in the tumor-bearing mouse model and also had therapeutic effects on metastatic tumors. Therefore, SDT combined immunotherapy can not only effectively treat the primary tumor, but also inhibit the recurrence of the tumor. The ICD induced by SDT plays an important role in the treatment of pancreatic cancer.

Over the past decade, the advent of immune checkpoint inhibitors (ICI) has made a transformational change for the field of immunooncology. For some tumor models, it has been found that blocking the receptor, programmed death 1 (PD-1), or its related ligand, programmed death ligand 1 (PD-L1) can effectively generate anti-tumor immunity [134, 139]. However, the limited immune activation and potential off-target side effects severely hinder its clinical application. To solve this issue, Huang et al. constructed cavitation-assisted endoplasmic reticulum targeting of nanodroplets (PMPS NDs) for SDT [140]. The nanodroplets were modified with cRGD peptide, a tumor vascular targeting peptide, and were able to effectively gather around tumor vascular endothelial cells after intravenous injection. Under the stimulation of US, the nanodroplets penetrated deeper, so that the sonosensitizer could accumulate and penetrate tumor tissue effectively. Subsequently, the released sonosensitizer targeted the endoplasmic reticulum, which can effectively improve the reaction range of ROS. Therefore, in the process of SDT, sonosensitizers can effectively induce endoplasmic reticulum stress and amplify ICD. In addition, the synergistic effect of anti-PD-L1 antibody combined with SDT has further improved the treatment effectiveness of orthotopic and distant pancreatic cancer.

**Fig. 10:**
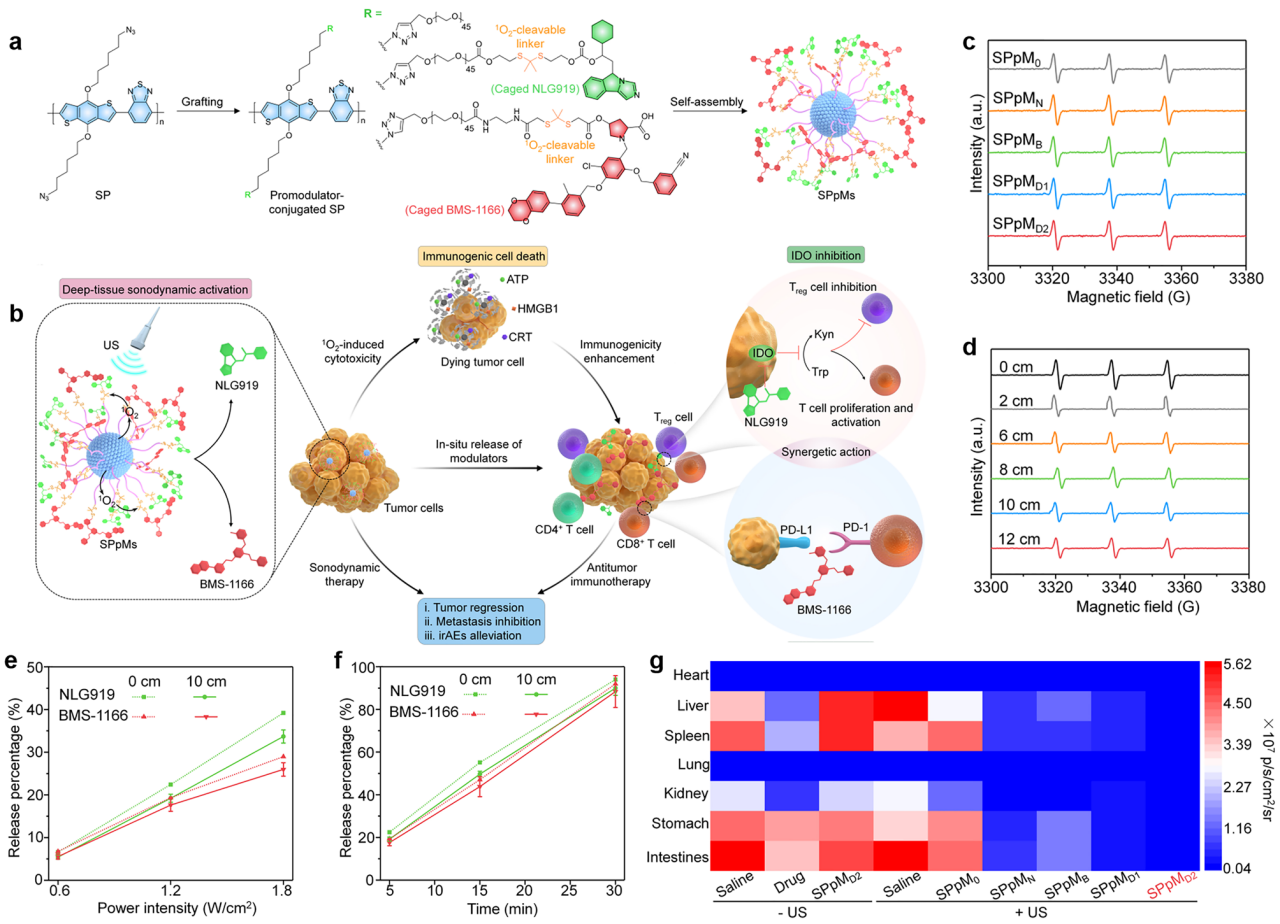
Sonoimmunotherapy induced by SPpMs for orthotopic pancreatic cancer. **a** Preparation of SPpMs. **b** Mechanism of synergistic therapy of SPpMs for sonodynamic and immunotherapy. Sonodynamic ^1^O_2_ generation properties of **c** SPpMs and **d** SPpM_D2_ covering pork tissues of different thicknesses. Release of NLG919 and BMS-1166 from SPpM_D2_ vary with changes in **e** ultrasonic power intensity and **f** treatment duration. **g** Bioluminescence signals of heart, liver, spleen, lung, kidney, stomach, and intestines in different treatment groups. Reproduced with permission [141]. Copyright 2023, John Wiley and Sons

Similarly, Pu et al. constructed US-activated semiconducting polymer pre-nanomodulators (SPpMs), consisting of a semiconductor polymer nanoparticle (SPN) as the core, which was surmounted with a polyethylene glycol (PEG) chain and connected to two immunomodulators (NLG919 and BMS-1166) through a singlet oxygen (^1^O_2_) response segment (Fig. 10a) [141]. Due to the excellent properties, SPpMs were used as a synergistic therapy of SDT and immunotherapy for orthotopic pancreatic cancer (Fig. 10b). Under US exposure, SPpMs effectively produced ^1^O_2_ (Fig. 10c), thereby directly killing tumor cells and inducing ICD effects even in deep tissues up to 12 cm deep (Fig. 10d). At the same time, the generated ^1^O_2_ initiated the break of the cleavable fragment, releasing NLG919 and BMS-1166 in deep tumors on demand, which induces antitumor immunity for a synergistic effect (Fig. 10e, f). Therefore, this deep-tissue US immunotherapy mediated by SPpMs could completely eradicate orthotopic pancreatic cancer in a living mouse model and effectively prevent tumor metastasis (Fig. 10g). In addition, the likelihood of immune-related adverse events was significantly reduced owing to the precise control of the immunotherapeutic effects in the tumor area. The study of the activatable nanosonosensitizer provides a new strategy for the precise treatment of orthotopic pancreatic cancer.

The proteolysis-targeting chimera (PROTAC)-mediated degradation of cancer proteins has become an effective strategy for cancer therapy [142–144]. Nevertheless, the clinical application of PROTACs has been retarded by issues, like poor water solubility, low bioavailability, and off-target adverse reactions [145, 146]. The tumor cell fragments produced by SDT act as tumor antigens, promoting lymphocyte infiltration into the tumor and inducing ICD of tumor cells [147]. However, ICD is accompanied by the release of large amounts of adenosine triphosphate, which can be converted into adenosine, which in turn binds to the adenosine 2A receptor on cancer and immune cells and exerts immunosuppressive effects [148]. This is accompanied by upregulation of PD-L1 leading to immune surveillance of tumor cells escaping.

To address these issues, Yuan et al. propose a PROTAC prodrug (NP_Ce6+PRO_), which can be activated by US for initiating effective ultrasonic immunotherapy in a spatiotemporal controlled manner [149]. NP_Ce6+PRO_ led to Ce6-mediated ROS production after US exposure at deep-tissue penetration, promoting SDT and inducing ICD. At the same time, the thioketone connectome could be cleaved in response to the generated ROS, causing PROTAC to be activated on demand at the tumor site to release the drug. This prodrug activation not only leads to the degradation of the target protein BRD4, but also inhibits the upregulation of the associated PD-L1 expression in SDT. In a mouse model of an orthotopic pancreatic tumor, NP_Ce6+PRO_ combined with US stimulation effectively inhibited tumor growth.

Likewise, owing to the complex TME, the effective treatment of pancreatic cancer is limited. Li et al. constructed an US-activated semiconductor polymer nanoreshaper (SPN_DN_H) to perform multiple remodeling of the TME of in situ pancreatic cancer to achieve effective treatment [150]. SPN_DN_H was consisting of a semiconductor polymer, a hydrogen sulfide donor, and an indoleamine 2,3-dioxygenase inhibitor (NLG919). They were enveloped in a hyaluronidase-modified and ^1^O_2_ response shell. After the accumulation of SPN_DN_H in the in situ pancreatic tumor site, SPN_DN_H effectively generated ^1^O_2_ through the SDT effect of semiconductor polymer under US irradiation, and the efficiency of SPN_DN_H was further improved since the degradation of the main content of hyaluronic acid in the TME, enhanced tumor accumulation of SPN_DN_H at tumor site, and alleviated hypoxia in TME. These amplified the production of ^1^O_2_ under US irradiation, leading to ICD. While the destruction of the ^1^O_2_ response component allowed NLG919 to be delivered to tumor tissue via acoustic activation. Infiltration of immune cells into the tumor was also improved by degrading the ECM matrix. This multiple remodeling strategy using SPN_DN_H elicited powerful anti-tumor immune effects, effectively inhibiting the growth of deep-tissue in situ pancreatic tumors and resistance to tumor metastasis in mouse models. This study provides an effective and accurate multi-remodeling strategy of TME for immunotherapy of deep in situ tumors.

In addition, the insufficient tumor infiltration of activated T cells also affected immunotherapy. For this, Xu et al. synthesized titanium diselenide (TiSe_2_) nanosheets with good stability, which exhibited a reliable nanosonosensitizer capable of inducing a large number of ROS with US irradiation under hypoxia and normoxia conditions to mediate SDT [151]. SDT-induced tumor-released TAAs mediated by TiSe_2_ nanosheets promoted ICD and favored dendritic cell maturation, cytokine secretion, and subsequent T-cell activation and infiltration into the tumor. SDT combined with anti-PD-1 immune checkpoint blocking therapy could effectively inhibit the growth of primary and distant tumors and prevent lung metastasis.

### Malignant Glioma

Malignant glioma, as a primary craniocerebral malignant tumor, has a poor prognosis, high recurrence rate, and mortality and seriously threatens human health and safety [152, 153]. The blood–brain barrier (BBB), which is composed of brain capillary endothelial cells, basal membrane, and glial cell foot process, seriously hinders drug delivery [154–156]. Compared with PDT, SDT not only has the characteristics of penetrating deep tissues, but also can reversibly open the BBB to enhance drug delivery to the brain [157].

Nanoscale metal-porphyrin coordination MOF not only has excellent stability and biocompatibility, but also the porous structure can enhance the interaction between US and the structure to enhance the generation of ROS and further promote the effect of SDT. Based on this, Chen et al. reported a heterogeneous structured nanoparticle, based on porphyrin MOFs, for the treatment of brain gliomas by SDT with amplified ROS destruction, guided by NIR IIb optical imaging penetrating the skull [158]. The heterostructured nanoagent was composed of luminescent descending nanoparticles (DSNPs) as the core and the iron-coordinated porphyrin MOF([PCN-224(Fe)]) as the epitaxial shell. The final DSNPs@MOF-sorafenib-lactoferrin nanoagent (DFMSL, ≈55 nm) was obtained by further loading the chemotherapy drug sorafenib (SRF) into the shell of MOF (entering the pore) and modified the surface of MOF with lactoferrin ligand. It had been proved that it can effectively inhibit tumors both in vivo and in vitro.

**Fig. 11:**
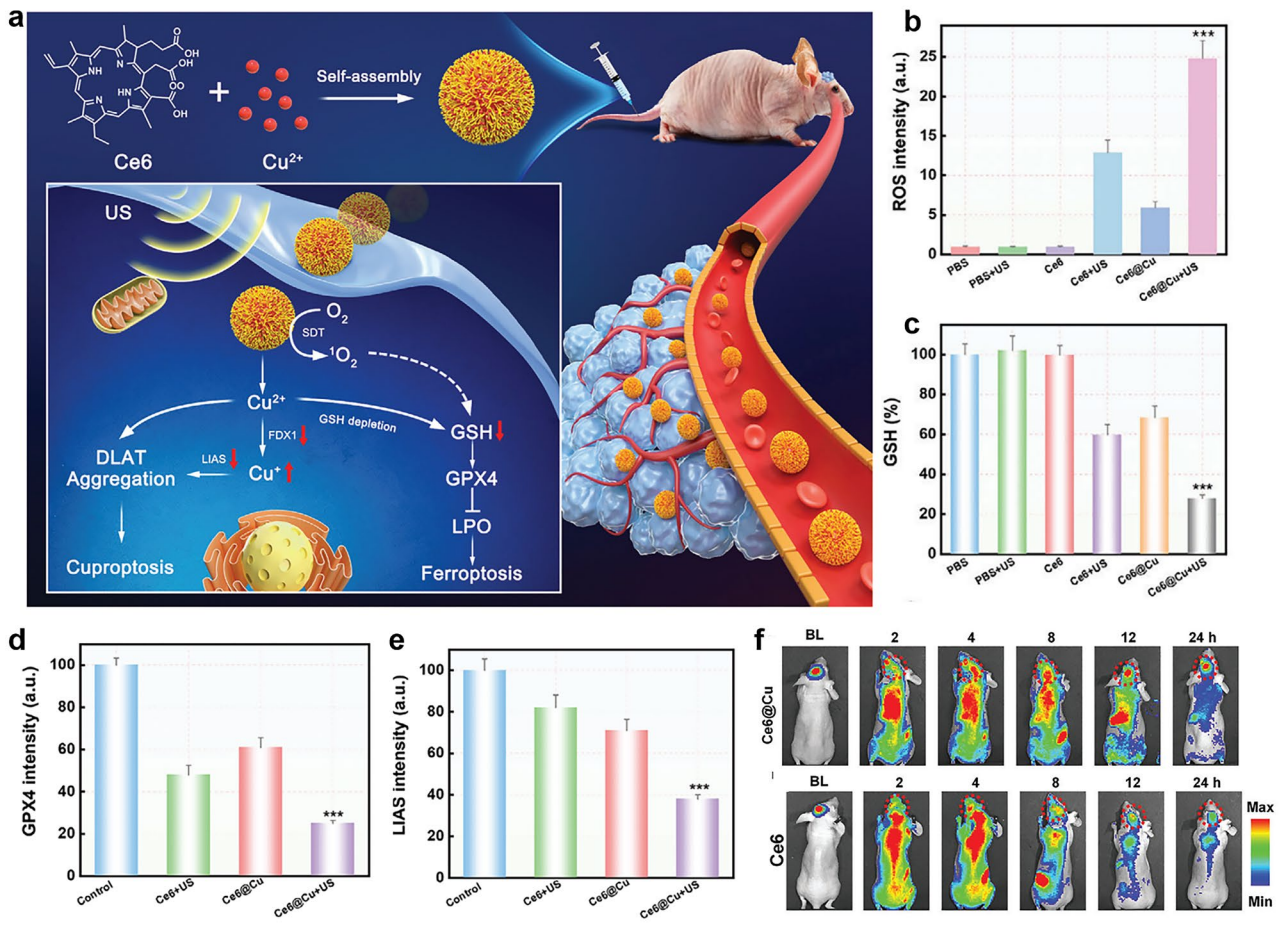
**a** Fabrication of Ce6@Cu NPs and sonodynamic-intensified cuproptosis and ferroptosis for orthotopic glioblastoma. **b** ROS generation in U87MG cells using DCFHDA as the fluorescence probe. **c** GSH level in U87MG cells treatment with different samples. The corresponding quantification of **d** GPX4 and **e** LIAS in U87MG cells treatment with differ samples. **f** In vivo fluorescence imaging of orthotopic U87MG-Luc tumor-bearing mice after intravenous injection of different samples. Reproduced with permission [159]. Copyright 2024, John Wiley and Sons

The increase of reductive GSH in tumor cells can clear excessive ROS produced by SDT, resulting in poor inhibition of tumor growth. In addition, depletion of GSH can induce the inactivation of GPX4 and accelerate lipid peroxidation, triggering large amounts of ferroptosis. Therefore, the construction of nanosonosensitizers with synergistic GSH depletion and ROS production capabilities is critical to inducing ferroptosis and conducting effective tumor therapy. For example, Kang et al. constructed a carrier-free nanoparticle (Ce6@Cu NPs) that was self-assembled by the coordination of Cu^2+^ ions with the sonosensitizer Ce6 [159]. The highly efficient sonodynamic effects of Ce6@Cu NPs synergically induced ferroptosis and cuproptosis in situ gliomas (Fig. 11a). After Ce6@Cu NPs were ingested by U87MG cells, Ce6@Cu NPs showed excellent sonodynamic effects under ultrasonic irradiation, producing a large amount of ^1^O_2_ (Fig. 11b). This led to the oxidation of polyunsaturated fatty acids, which then triggered deadly lipid peroxidation. In addition, Ce6@Cu NPs effectively depleted overproduced reduced GSH in tumor cells (Fig. 11c), resulting in the inactivation of GPX4 (Fig. 11d), accelerated lipid peroxidation, and irreversible ferroptosis. Due to the reaction of Cu^2+^ with reduced GSH, Cu^+^ concentration in U87MG cells was significantly increased, resulting in down-regulation of ferredoxin-1 and LIAS expression (Fig. 11e). This process significantly promoted oligomerization of the lipacylated dihydrolipoamide S-acetyltransferase, triggering protein toxic stress and ultimately cuproptosis of the cells. Importantly, Ce6@Cu NPs demonstrated a satisfactory ability to penetrate the BBB and were significantly enriched in situ U87MG-Luc glioblastoma (Fig. 11f). The synergistic induction of ferroptosis and cuproptosis by SDT of Ce6@Cu NPs had been demonstrated in vitro and in vivo with few side effects. This study not only provides a carrier-free nanosonosensitizer for the treatment of malignant glioma, but also affords a promising tumor treatment strategy based on synergistic ferroptosis and cuproptosis.

Because of the presence of BBB, it is hard for chemotherapy drugs to enter the brain. In addition, the resistance of glioma cells to chemotherapy drugs has been increased by macrophage/autophagy, which greatly reduces the effectiveness of chemotherapy. The cavitation effect of US can instantly and reversibly destroy the BBB and promote drug entry into the brain, providing a new strategy for effective drug delivery across the BBB. Hydroxychloroquine (HCQ), as the only clinically available autophagy inhibitor, has caused serious side effects and limited clinical application. For instance, Wang et al. designed an intelligent "all-in-one" nanosonosensitizer platform (named ACHL) by loading the sonosensitizer Ce6 and the autophagy inhibitor HCQ into angiopep-2 peptide-modified liposomes [160]. ACHL could not only minimize the toxic side effects of HCQ but also open the BBB instantaneously under the mediation of US, effectively delivering drugs into the brain. Subsequently, under the action of US, the nanosonosensitizer platform generated ROS for SDT, while the loaded HCQ was released into glioma cells to inhibit autophagosome degradation, synergically inhibited the tumor growth, and improved the therapeutic effect. The design of ACHL provides new insights for the treatment of brain tumors. In addition, they also constructed nanosonosensitizer (iRGD-Lipo-DVDMS) by loading DVDMS into iRGD (internalizing RGD, CRGDKGPDC)-modified liposomes [161]. As iRGD is a tumor-homing peptide, iRGD-Lipo-DVDMS had the ability of targeting tumors, thus significantly enhancing the accumulation of iRGD-Lipo-DVDMS at the tumor site. Under the irradiation of US, the ROS generated by iRGD-Lipo-DVDMS could effectively inhibit orthotopic glioma, showing a remarkable anti-tumor effect.

### Breast Cancer

TNBC has a dense matrix of rich collagen fibers, which forms a barrier that restricts the delivery of nanomaterials to the tumor parenchyma and hinders tumor invasion by cytotoxic T lymphocytes (CTL) [162, 163]. Therefore, developing a strategy that can promote the penetration of therapeutic nanomaterials and CTLS in tumors is of great significance for the treatment of TNBC. SDT as a burgeoning tumor treatment strategy with non-invasive, highly controllable, and deep penetration provides a new approach to the treatment of TNBC. In terms of clinical application, Inui et al. reported on a 55-year-old woman with recurrent breast cancer [164]. After modified Tin Chlorin e6 and 5-ALA-mediated SDT, macrophage activation (Gc protein-derived macrophage activating factor, GcMAF), and hormone therapy (exemestane), right lung pleural effusion and intrapleural nodular tumor disappeared completely. The development of new low band gap nanosonosensitizers is necessary to raise the production of ROS and thus enhance the anti-tumor efficacy. In addition, the use of nanosonosensitizers to overcome the tumor matrix barrier and penetrate into the tumor interior is an attractive tactics to improve the outcome of SDT tumor therapy.

For example, the group of Tao designed a denaturation and penetration strategy and used stannous sulfide nanoparticles (SnSNPs) as nanosonosensitizer to treat TNBC [165]. SnSNPs nanoparticles had a narrow band gap (1.18 eV) and could efficiently separate electron and hole pairs under ultrasonic activation to generate ROS. In addition, SnSNPs nanoparticles also had mild photothermal properties, which could in situ denature collagen in tumor matrix under NIR light, thus promoting the deep penetration of nanoparticles and immune cells into the tumor to further exert anti-tumor effects. This strategy significantly improved the efficacy of anti-tumor SDT and enhanced the anti-tumor immune response. This treatment strategy achieved significant antitumor effects in a mouse model. The study opens up an entirely new avenue to enhance SDT and anti-tumor immune responses using denaturation and penetration strategies, providing a potential combined SDT-immunotherapy approach to the field of cancer nanomedicine.

Since the synergistic anti-cancer efficiency of multiple therapies is better than that of single therapy, Gao et al. prepared a carrier-free nanosonosensitizer OC based on the self-assembly of natural hydrophobic anti-cancer drug oleanolic acid and the photosensitizer Ce6, realizing the synergistic treatment of chemotherapy, PDT and SDT [166]. OC had good dispersion in aqueous solution, which could not only promote the uptake of nanosonosensitizer but also facilitate the penetration of nanosonosensitizer in tumors. Then, the antitumor effect of OC was studied on an orthotopic 4T1 breast tumor-bearing mouse model, and the antitumor effect was significant.

Similarly, Li and their coworkers designed polylactic glycolic acid nanoparticles (CHINPs) loaded with superparamagnetic iron oxide (SPIO) and hematoporphyrin monomethyl ether (HMME) and modified by 4T1 cancer cell membranes [167]. Because the modification of the cell membrane endows CHINPs with homologous targeting ability, the accumulation of CHINPs in the tumor region has been enhanced. HMME, as a sonosensitizer, could produce ROS to kill tumor cells under ultrasonic irradiation, while SPIO, as a photothermal agent, could accelerate tumor blood flow and O_2_ concentration to enhance the efficiency of SDT. Besides, PTT and SDT could further induce an immune response to inhibit tumor metastasis. This multi-mode collaborative anti-tumor strategy could not only eradicate tumors in situ and stimulate systemic immune response but also has the ability of photoacoustic, magnetic resonance, and photothermal imaging to provide imaging guidance for tumor precision treatment. Substantial animal experiments had indicated that the tumor cell fragments, generated during SDT via inducing tumor cell death by activating sonosensitizers to produce ROS through US, could serve as a source of tumor antigens, which can trigger an immune response from the host.

**Fig. 12:**
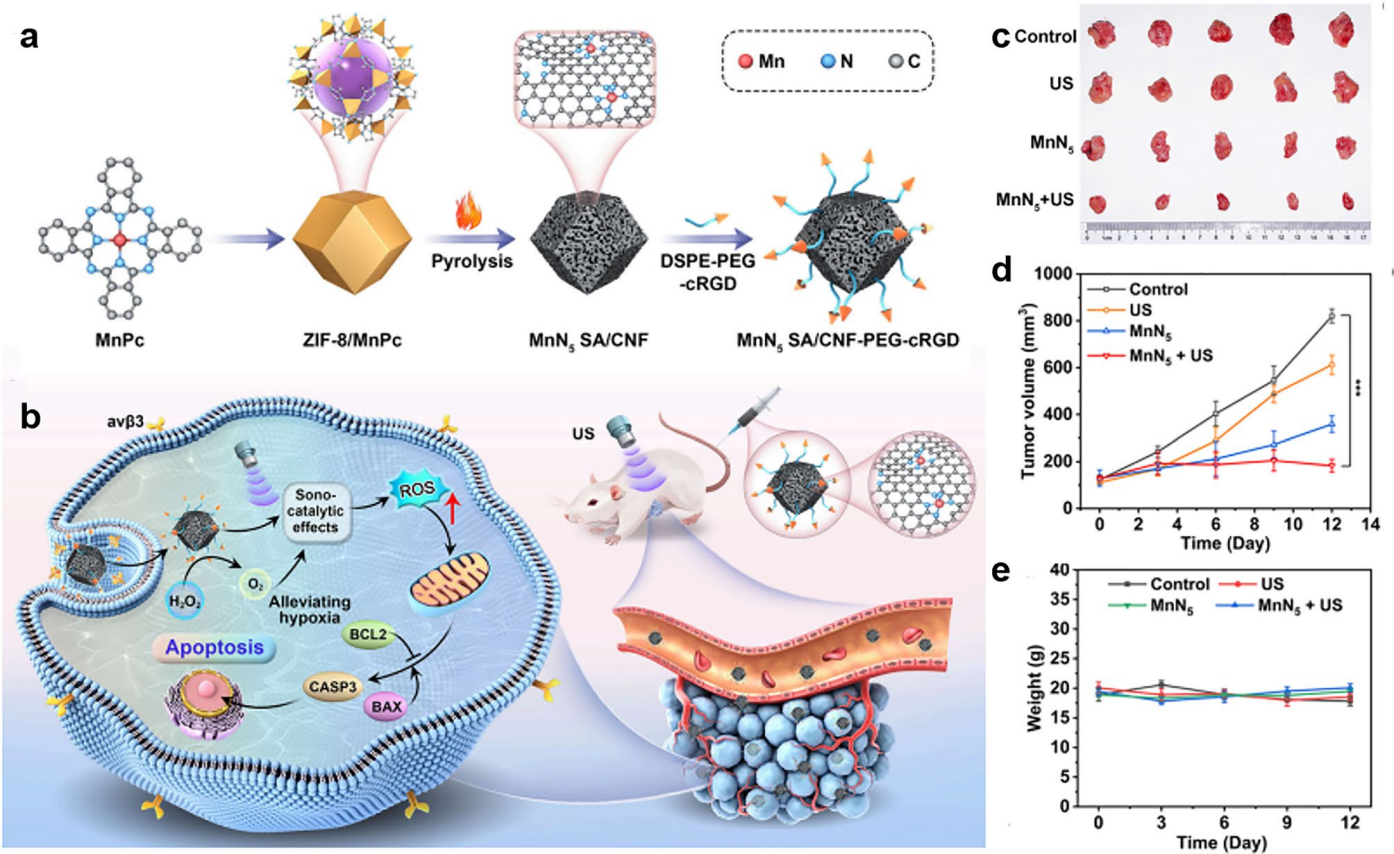
MnN5 SA/CNF for the treatment of orthotopic breast cancer. **a** Schematic diagram of fabricating MnN5 SA/CNF. **b** MnN5 SA/CNF for SDT. **c** Optical photographs of tumors, **d** tumor volume growth curves, and **e** body weight after various treatments [168]. Copyright 2024, American Chemical Society

Although SDT has emerged as a promising non-invasive tumor therapy, its therapeutic efficiency has been hampered by the lack of US-induced ROS and the hypoxic microenvironment of the tumor. Pan et al. utilized phthalocyanine-mediated pyrolysis strategy to develop a carbon nanoframe-confined N-coordination manganese single-atom sonosensitizer (MnN5 SA/CNF) with pentacoordinate structure (Fig. 12a) [168]. Compared with traditional tetracoordinate manganese (MnN4 SA/CNF) and MnO_2_, MnN5 SA/CNF had superior sonodynamic efficiency and significantly higher multi-enzyme-like catalytic activity, which effectively overcame the hypoxic tumor microenvironment, thus significantly improving the efficiency of SDT (Fig. 12b). This was mainly attributed to the optimized coordination structure and the defect-enhanced cavitation effect, and the pentacentate structure reduced the d-band center of Mn from − 0.547 to − 0.829 eV, which enhanced the desorption ability of oxygen-containing intermediates, thus accelerating the catalytic process. Finally, MnN5 SA/CNF was applied in the mouse model of orthotopic breast cancer. Figure 12c, d verifies that the effective tumor inhibition achieved up to 94.43%, while Fig. 12e verifies MnN5 SA/CNF had almost no damage to normal tissues. This coordinated regulatory strategy for sonosensitizers represents a major advance in SDT.

## Conclusions and Perspectives

To sum up, as an emerging treatment mode, SDT is an extremely promising treatment modality because of its advantages, including non-invasiveness, spatiotemporal controllability, high therapeutic efficacy, deep-tissue penetration, and promoting drug delivery. Therefore, SDT has been extensively studied in recent years. However, the mechanism of SDT has not been clearly explained. At present, the proposed mechanisms mainly include ultrasonic cavitation effect, and mechanical and thermal damage. The most widely accepted mechanism is the triggering of ROS production by sonosensitizer or cavitation effect. Therefore, it is necessary to further summarize the mechanism of SDT to provide a direct theoretical basis for improving SDT. Sonosensitizers are the core components of SDT. Although various sonosensitizers have been developed and indicated the feasibility of SDT in biomedical applications, its clinical application faces many challenges. Nanosonosensitizers have provided help for overcoming the obstacles of the TME and can be tailored and modified in response to various tumor characteristics, allowing sonosensitizers to better adapt to the specific needs of treatment. For instance, the BBB poses a significant obstacle in the treatment of glioblastoma. Fortunately, the EPR effect of nanomaterials can effectively facilitate the transport of drugs across the BBB, thereby elevating their concentration within the brain. Secondly, specific modifications can be introduced on the surface of the nanosonosensitizers, such as linking targeting ligands (like transferrin receptor antibodies). These ligands can selectively bind to their corresponding receptors on the BBB, enabling targeted delivery of the sonosensitizers. In this paper, the application of nanotechnology-assisted sonosensitizers in SDT in the past five years is reviewed. Different strategies for enhancing tumor accumulation, improving safety with image-guiding therapy, reducing endogenous antioxidants such as GSH, and overcoming hypoxia have been carried out. In addition, SDT has a deeper tissue penetration capacity than PDT. Therefore, it has better performance in different cancer types. Table 2 summarizes the treatment cases of SDT for different types of orthotopic tumors and further optimizes the treatment combined with other treatment modes.

**Table 2:** Summary of SDT for the orthotopic tumor treatment

Therapeutic modality	Nanosonosensitizer	Strategy	Orthotopic tumor model	US power (in vivo)	References
SDT	Ti-TCPP MOF	Directly targeted to the nuclei	Pancreatic carcinoma	0.5 W cm^−2^	[124]
SDT	Sonosensitizer-Free	Respond to the acidic TME and local US to release CO_2_ bubbles, enhancing inertial cavitation	Pancreatic cancers	1 W cm^−2^, 1 MHz, and 50% duty cycle	[125]
SDT + CDT	AIPH@Cu-MOF NPs	Oxygen-independent free radical generation	Orthotopic pancreatic	0.5 W cm^−2^, 1 MHz, 50% duty cycle, 2 min	[132]
SDT + immunotherapy	PMPS NDs	Cavitation-assisted ER-targeted	Pancreatic cancer	1.0 W cm^−2^; Duty cycle: 20%; Times: 5 min	[140]
SDT + immunotherapy	NP_Ce6+PRO_	PROTAC prodrug in a spatiotemporally controllable manner	Pancreatic tumors	1.0 MHz, 1.0 W cm^−2^, 50% duty cycle, 5 min	[149]
SDT + immunotherapy	SPN_DN_H	Multiply remodel TME for effective and precise immunotherapy of deep-tissue orthotopic tumors	Pancreatic cancer	1.0 W cm^−2^, 1.0 MHz, 50% cycle	[150]
SDT + immunotherapy	SPpMs	^1^O_2_-cleavable segments to allow in situ release of immunomodulators in tumors	Orthotopic pancreatic cancer	1.2 W cm^−2^, 50% duty cycle, 1.0 MHz, 5 min	[141]
SDT	MOF, PCN-224(Fe)	Chemotherapeutics effectively inhibit GSH synthesis, precise optical imaging	Intracranial Glioma	1 W cm^−2^, 1.0 MHz, 50% duty cycle	[158]
SDT + chemotherapy	ACHL	Ultrasonic pulse promoted the ACHL into the reversibly opened BBB	Gliomas	0.6 W cm^−2^	[160]
SDT	Ce6@Cu NPs	Sonodynamic-triggered combination of cuproptosis and ferroptosis	U87MG-Luc glioblastoma	1 W cm^−2^ for 10 min	[159]
SDT	iRGD-Lipo-DVDMS	Loaded into the targeting liposomes carrier-free nanosensitizer	Orthotopically implanted C6	0.6 W cm^−2^	[161]
SDT + PDT + chemotherapy	OC	Carrier-free nanosensitizer	4T1 breast tumor	1 W cm^−2^, 90 s, 100% duty cycle	[166]
SDT + PTT + Immunotherapy	CHINPs	Homologous tumor-targeting property multimodal imaging-guided triple therapeutic nanoplatforms	4T1 tumor	2.0 W cm^−2^, 1 MHz, 50% duty cycle	[167]
SDT/PTT/immunotherapy	MnN5 SA/CNF	Liposomes with high biocompatibility	4T1 tumor	1.0 W cm^−2^, 1 MHz, 50% duty cycle	[168]

**Scheme 2:**
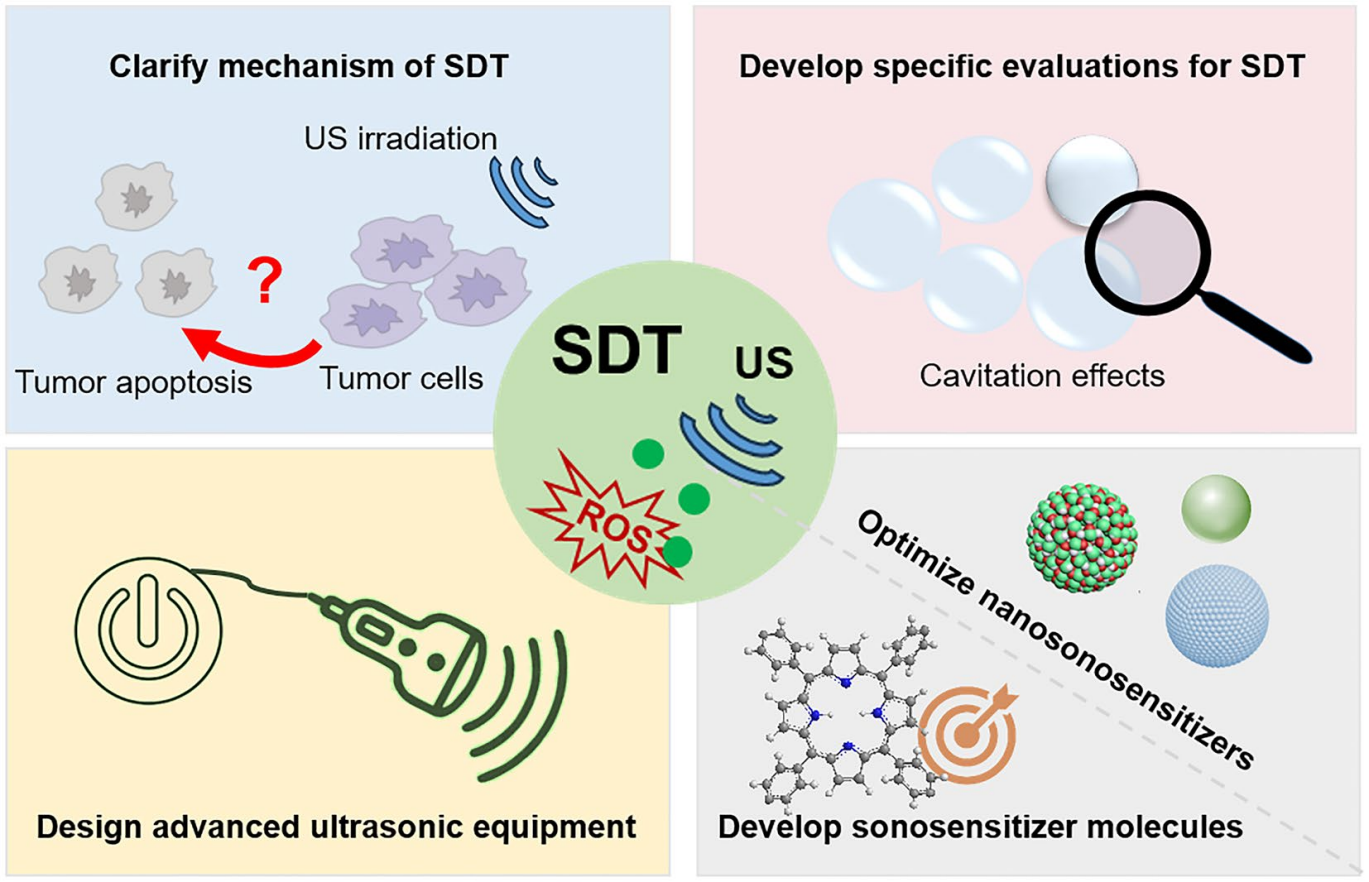
Schematic illustration of the perspectives of SDT in the future

Although researchers have made considerable efforts to design and fabricate safe and effective novel sonosensitizers, and have yielded numerous promising outcomes in preclinical studies, there remain several pivotal issues and challenges that must be tackled for its successful translation into clinical practice. These include the mechanism of SDT, the evaluation of SDT, the toxic side effects of sonosensitizer molecules, ultrasonic equipment, and complex preparation techniques (Scheme 2). Therefore, the future research should more focus on:

1. Clarify the mechanism of SDT, including biological toxicity and therapeutic efficiency. This will contribute to the construction of realistic SDT-mediated therapies and provide direction for the rational design of sonosensitizers in the future.
2. Develop specific evaluations for SDT. At present, there is no comprehensive method to evaluate the effect of SDT, which is mainly estimated by measuring the ability to produce ROS, and there is no comparable assessment approach for cavitation effects. Therefore, it is important to develop a set of guidelines for the evaluation of sonosensitizers and SDT-related properties.
3. Develop new sonosensitizer molecules. At present, the most widely studied sonosensitizer molecules are still photosensitizers, but most of these molecules can cause serious phototoxicity and adverse side effects. Therefore, it is urgent to design and synthesize safe and efficient sonosensitizers with low phototoxicity, high sensitivity, and high tumor selectivity.
4. Design advanced ultrasonic equipment. The successful implementation of SDT depends on ultrasonic equipment, yet current ultrasonic devices are solely suitable for the study of mice in vivo and in vitro, rather than for clinical treatment in humans. Due to the differences in tissues, bones, and other aspects between humans and mice, clinical treatment in humans requires the US with higher power. Advanced equipment is capable of optimizing ultrasonic parameters to ensure its feasibility and practicality in clinical practice. Therefore, more advanced US equipment is needed to perform multiple functions simultaneously, which is crucial for enhancing the efficacy of SDT.
5. Optimize nanosonosensitizers. Although nanomaterials can enhance the therapeutic efficacy of sonodynamic treatment through strategic modifications, they still face several critical technical challenges in clinical translation. Firstly, the stability of nanomaterials in biological environments is affected by biomolecules, which in turn impacts their sonosensitive effectiveness. Additionally, safety assessment is an indispensable step prior to the clinical application of nanomaterials. However, most nanosensitizers have complex structures and lack biocompatibility and biodegradability, significantly impeding their large-scale production and further application. Therefore, optimizing the simplicity of their manufacture, enhancing the stability and biological safety of nanomaterials, and achieving the most effective and mutually reinforcing synergistic effects are conducive to further advancing their clinical applications.

With the cutting-edge discovery and innovation of science, we believe that the above problems will be solved in the near future, and SDT will exhibit an important role in the clinical treatment of cancer.
